# Genome-scale reconstruction and system level investigation of the metabolic network of *Methylobacterium extorquens *AM1

**DOI:** 10.1186/1752-0509-5-189

**Published:** 2011-11-10

**Authors:** Rémi Peyraud, Kathrin Schneider, Patrick Kiefer, Stéphane Massou, Julia A Vorholt, Jean-Charles Portais

**Affiliations:** 1 Institute of Microbiology, ETH Zürich, 8093 Zürich, Switzerland; 2 Université de Toulouse; INSA, UPS, INP; LISBP, 135 Avenue de Rangueil, F-31077 Toulouse, France; 3 INRA, UMR792, Ingénierie des Systèmes Biologiques et des Procédés, F-31400 Toulouse, France; 4 CNRS, UMR5504, F-31400 Toulouse, France

## Abstract

**Background:**

Methylotrophic microorganisms are playing a key role in biogeochemical processes - especially the global carbon cycle - and have gained interest for biotechnological purposes. Significant progress was made in the recent years in the biochemistry, genetics, genomics, and physiology of methylotrophic bacteria, showing that methylotrophy is much more widespread and versatile than initially assumed. Despite such progress, system-level description of the methylotrophic metabolism is currently lacking, and much remains to understand regarding the network-scale organization and properties of methylotrophy, and how the methylotrophic capacity emerges from this organization, especially in facultative organisms.

**Results:**

In this work, we report on the integrated, system-level investigation of the metabolic network of the facultative methylotroph *Methylobacterium extorquens *AM1, a valuable model of methylotrophic bacteria. The genome-scale metabolic network of the bacterium was reconstructed and contains 1139 reactions and 977 metabolites. The sub-network operating upon methylotrophic growth was identified from both *in silico *and experimental investigations, and ^13^C-fluxomics was applied to measure the distribution of metabolic fluxes under such conditions. The core metabolism has a highly unusual topology, in which the unique enzymes that catalyse the key steps of C1 assimilation are tightly connected by several, large metabolic cycles (serine cycle, ethylmalonyl-CoA pathway, TCA cycle, anaplerotic processes). The entire set of reactions must operate as a unique process to achieve C1 assimilation, but was shown to be structurally fragile based on network analysis. This observation suggests that in nature a strong pressure of selection must exist to maintain the methylotrophic capability. Nevertheless, substantial substrate cycling could be measured within C2/C3/C4 inter-conversions, indicating that the metabolic network is highly versatile around a flexible backbone of central reactions that allows rapid switching to multi-carbon sources.

**Conclusions:**

This work emphasizes that the metabolism of *M. extorquens *AM1 is adapted to its lifestyle not only in terms of enzymatic equipment, but also in terms of network-level structure and regulation. It suggests that the metabolism of the bacterium has evolved both structurally and functionally to an efficient but transitory utilization of methanol. Besides, this work provides a basis for metabolic engineering to convert methanol into value-added products.

## Background

Methylotrophs are microorganisms able to grow on reduced C1 compounds such as methane and methanol as sole source of carbon and energy. Methylotrophy has gained increasing interest over the past decade for both basic and applied purposes, since methanol can be produced from diverse renewable sources and represents a valuable feedstock for biotechnological applications [1,2]. Recent progress in the biochemistry, genetics, genomics, and physiology of methylotrophic bacteria has shown that methylotrophy is a phenomenon much more widespread and versatile than initially assumed [3]. The phylogenetic distribution of methylotrophy is broad and spans over a range of phyla and genera [3]. Methylotrophic bacteria are adapted to various lifestyles and ecological niches (soil, water sediments, plant roots, phyllosphere), and are involved in a number of important biological or biogeochemical processes, in particular the global carbon cycle. Methylotrophy encompasses diverse metabolic capabilities or behaviors that were used to classify them, e.g. obligate vs facultative methyltrophy, heterotrophic vs autotrophic growth, aerobic vs anaerobic metabolism. Such genetic and biochemical diversity may explain the ecological success of methylotrophs, which can represent dominant microbial populations in specific environments, e.g. plant leaves [4].

From the biochemical point of view, methylotrophy relies on specific pathways (C1 pathways) ensuring all growth requirements. Energetic requirements are enabled by dissimilation pathways, which involve a series of three basic steps: i) the oxidation of primary C1 substrates - methanol, methylamine - typically into the toxic intermediate formaldehyde, ii) the oxidation of the latter compound into formate, and iii) formate oxidation into carbon dioxide (CO_2_). In addition, alternative dissimilation mechanism exist in which formate is not involved. The assimilation of C1-units can be achieved by different mechanisms starting from either formaldehyde (ribulose-monophosphate pathway, RuMP), CO_2 _(Calvin-Benson-Bassham cycle, CBB) or methylene-tetrahydrofolate (Me-THF) + CO_2 _(serine cycle). Novel enzymes, biochemical mechanisms, and metabolic pathways have been discovered, resulting in a more complete description of methylotrophic pathways and their diversity. Interestingly, these findings do not modify our current view of the general organization of C1 pathways, but show that many more alternative enzymes or pathways than initially assumed carry out each of the basic steps of methylotrophy. Moreover, a growing number of newly discovered methylotrophs have been investigated, showing that different combinations of the various pathways can be found in nature, and leading to the concept of a modular metabolism [5].

Considerable progress was made in the understanding of the biochemistry and physiology of methylotrophy, and valuable insights were obtained regarding the organization and operation of central carbon metabolism [6-8]. However, a complete, system-level description of methylotrophy metabolism is currently lacking. The comprehensive understanding of bacterial physiology requires a detailed knowledge of the complete metabolic potential of the studied organism, but such knowledge is currently missing and no genome-scale metabolic network has been so far established for any methylotrophic bacterium. In consequence, little is currently known about the network-scale organization of methylotrophy, the specific properties of methylotrophic networks, and how the methylotrophic capacity emerges from this organization, especially in facultative representatives. Among the recently sequenced organisms, the Alphaproteobacterium *Methylobacterium extorquens *AM1 is a major model of methylotrophic bacteria. This facultative methylotroph is able to grow on C1- but also on multicarbon (C2-C4) compounds. *Methylobacterium *spp. are part of the abundant population of bacteria systematically found in the phyllosphere [4,9], where they benefit from plant-derived methanol [10,11]. The biochemistry, genetics, and physiology of *M. extorquens *AM1 has been extensively investigated, and allowed the discovery of major methylotrophic pathways, including the serine cycle [12-14], the tetrahydromethanopterin (H_4_MPT)-dependent pathway for formaldehyde oxidation [15,16], and of a number of novel enzymes or enzyme functions (e.g., pyrroloquinoline quinine (PQQ)-dependent methanol dehydrogenase, formaldehyde activating enzyme, methylene-H_4_MPT dehydrogenases coupled to pyridine nucleotides, and formyl-methanofuran hydrolase). The central metabolic pathways were progressively unraveled, and the application of novel experimental strategies - metabolic modeling, metabolic flux analysis, omics technologies - provided valuable insights into the topology and operation of central carbon metabolism [6-8,17]. Finally, the pathway for methanol assimilation in *M. extorquens *AM1 was recently completed with the discovery of the ethylmalonyl-CoA pathway (EMCP), an alternative to the glyoxylate cycle for the synthesis of glyoxylate, which encompasses an unusual series of 12 reactions where intermediates are all Coenzyme A (CoA) esters [18,19]. Finally, the genome of this bacterium was recently sequenced and annotated [20], providing inevitable information for genome-scale investigations.

In this work, we report on the integrated, system-level investigation of the metabolic network of the facultative methylotroph *M. extorquens *AM1, as a valuable model of methylotrophic bacteria. In order to obtain a comprehensive understanding of the biochemistry and physiology of this bacterium, we first evaluated its complete metabolic potential by compiling current biochemical knowledge and genome annotation data into a comprehensive genome-scale representation of the metabolic network. Then, we performed both *in silico *and experimental investigations to identify the subnetwork operating during methylotrophic growth, in order to analyze structurally and functionally the system-level organization of methylotrophy with an emphasis on the properties of the organization of central carbon metabolism.

## Results

### Metabolic network reconstruction

The genome-scale (GS) metabolic network of *M. extorquens *AM1 was reconstructed according to previously established guidelines [21]. The details of the process are given in material and methods and are schematically shown in Additional file 1. Briefly, the GS metabolic network was obtained by integrating relevant information collected from i) genome annotation [20], ii) published physiological, genetic and biochemical studies in *M. extorquens *AM1 and closely related organisms iii) biochemical information contained in databases [22-24], vi) complementary investigations (biomass quantification), and intensive refinement (Additional file 2, 3). The chemical composition of *M. extorquens *AM1 cell was determined experimentally or taken from available literature (Table 1 and Additional file 4) and used to define the biosynthetic needs and corresponding pathways.

**Table 1:** Chemical content and physiological parameters of *M. extorquens *AM1 cells growing on methanol

Macromolecule	% Cell Dry Weight ± σ		Data source	Organism source
**Protein**	**59.13 ± 2.11**		This study	*M. extorquens *AM1
**Carbohydrate**	**16.43 ± **1.09		This study	*M. extorquens *AM1
Rhamnose (polymer)	8.92 ± 0.92		This study	*M. extorquens *AM1
Glucose (polymer)	5.62 ± 0.52		This study	*M. extorquens *AM1
Trehalose	1.22 ± 0.20		This study	*M. extorquens *AM1
Glucosamine (polymer)	0.09 ± 0.67		This study	*M. extorquens *AM1
**RNA**	**8.20 ± 0.68**		This study	*M. extorquens *AM1
**Fatty acid**	**4.95 ± 0.29**		This study	*M. extorquens *AM1
**DNA**	**3.00 **-		Neidhart *et al.*; GC content: Vuilleumier et al. (2009)	*E. coli*
**PHB**	**2.36 ± 0.05**		This study	*M. extorquens *AM1
**Polyamine**	**0.40 **-		Neidhart *et al.*	*E. coli*
**Carotenoid**	**0.023 **-		Konovalova *et al. *(2007)	*M. extorquens *AM1
**Intracellular metabolites**	**2.64 **-		Kiefer *et al. *(2008); Guo *et al. *(2006); Guo *et al. *(2007); Vorholt *et al. *(1998); Crowther *et al. *(2008)	*M. extorquens *AM1
**Inorganic ions**	**1.01 **-		Neidhart *et al.*	*E. coli*
**Cofactors**	**0.22 **-		Neidhart *et al.*	*E. coli*

**SUM**	**98.36**			
**Physiological parameters**	**value ± σ**	**units**	**Data sources**	**Organism source**

**Growth rate**	**0.168 ± 0.003**	h^-1^	This study	*M. extorquens *AM1
**Specific methanol uptake rate**	**15.0 ± 0.25**	mmol.g^-1^.h^-1^	This study	*M. extorquens *AM1
**Specific proton production rate**	**0.22 ± 0.01**	mmol.g^-1^.h^-1^	This study	*M. extorquens *AM1
**Growth-associated ATP maintenance**	**59.81 **-	mmol.g^-1^	Neidhart *et al.*	*E. coli*
Macromolecular building costs	**26.65 **-	mmol.g^-1^	This study	*M. extorquens *AM1
**Non-Growth-associated ATP maintenance**	**9.5 **-	mmol.g^-1^.h^-1^	Rokem *et al. *(1978)	*Methylobacterium*

The refined reconstruction was converted into a mathematical model using CellNetAnalyser [25] and the network was checked for self-consistency and curated to allow biosynthesis of all cell components from each of the 12 carbon sources established for *M. extorquens *(Additional file 5). In addition, flux balance analysis (FBA) was used to calculate the theoretical maximum growth yields for each carbon source. For methanol and succinate, the theoretical maximum growth rates could also be calculated and were in agreement with published data (Additional file 5), showing the consistency of the network with experimental observations. Last, the capability of the GS network to explain the oxidation of carbon compounds [26] was validated (Additional file 5).

The final GS network (iRP911) contained 1139 unique reactions and 977 metabolites, and was based on a gene-to-protein-to-reaction (GPR) association network that included 911 genes encoding 761 proteins (Table 2, Additional file 2, 3). The confidence in the network information was established by scoring the evidence currently available for each reaction [21]. The confidence scores ranged from 0 (lowest) to 4 (highest), with the latter being assigned to a reaction with direct evidence for both gene product function and biochemical reaction (Table 2). The average confidence score over the final network score was 2.1.

**Table 2:** Properties of the genome-scale (iRP911) and methylotrophic networks reconstructed for *M. extorquens *AM1

Properties		GS network	%	Methylotrophic network		% of GS network
Biochemically unique reactions		1139		717		62.9%
	Reversible reactions	578	50.7%	340		58.8%
Metabolites		977		722		73.9%
Genes		911		706		77.5%
Enzymes		761		595		78.2%
	Protein complexes	83	10.9	65		78.3%
						
**Confidence score of reactions (GS network)**			**Number**	**%**	**Data sources**	

4	Experimental evidence for enzyme activity		54	4.7%	Biochemical data	
4	Spontaneous reaction		15			
3	Experimental evidence for gene function		28	2.5%	Genetic data	
2	Genome annotation		856	75.2%	Genomic data	
2	Evidence from physiology (Transport)		127	11.2%	Physiological data	
1	Hypothetical reaction required for modeling		59	5.2%	Modeling data	
**2.1**	**Average confidence score of the network**					

### Main features of the genome-scale metabolic network of *M. extorquens *AM1

**Figure 1:**
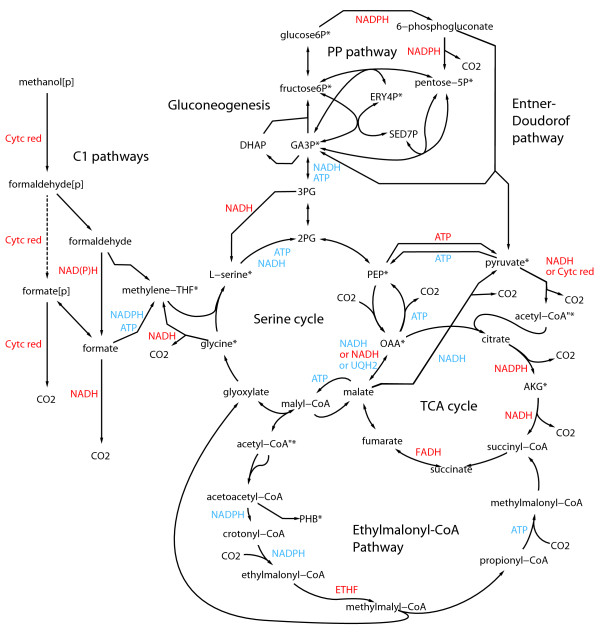
**Central carbon metabolism of *M. extorquens *AM1**. Precursors of biomass components are labeled with an asterisk (*). Some metabolites were duplicated on the map for clearer visualization, and are indicated with ". Cofactors used as substrate or products are indicated in blue and red, respectively. GA3P, glyceraldehyde-3-phosphate; 3PG, 3-phosphoglycerate; 2PG, 2-phosphoglycerate; PEP, phosphoenol-pyruvate; OAA, oxaloacetate; 3HBCOA, 3-Hydroxy-butyryl-Coenzyme A; E4P, D-erythrose-4-phosphate; AKG, α-ketoglutarate; SED7P, sedoheptulose-7-phosphate; cyt c red: reduced cytochrome c. Dotted arrows represent uncertain reactions.

*M. extorquens *AM1 is a facultative methylotroph able to utilize a relatively narrow range of substrates. The ability to grow on C3 and C4 organic acids - e.g. lactate, pyruvate, succinate or malate - relies on the presence of common metabolic pathways, which include the tricarboxylic acid (TCA) cycle, anaplerotic pathways, gluconeogenesis, pentose-phosphate pathway (PPP), and Entner-Doudoroff (ED) pathway (Figure 1). Growth on C1 compounds relies on specific metabolic pathways that were resolved for this bacterium in the past 50 years [5,16]. The first step is the oxidation of primary C1 substrates - e.g. methanol - to formate via methanol dehydrogenase and the H_4_MPT-dependent C1 pathway. Formate is a key branch-point to trigger the flow of carbon between dissimilation and assimilation. Dissimilation is achieved by oxidation of formate into CO_2_. The assimilation of C1-units requires the conversion of formate into methylene tetrahydrofolate (Me-THF), since the spontaneous condensation of formaldehyde with THF was demonstrated not to be significant [7]. In the serine cycle, Me-THF is condensed with a C2 compound - generated from glyoxylate - to build C3 compounds such as 2-phosphoglycerate and phosphoenolpyruvate (PEP), which are further carboxylated to form oxaloacetate (OAA) and other C4 intermediates. The continuous operation of the serine cycle requires the operation of the recently discovered EMCP which allows glyoxylate regeneration and involves CO_2 _fixation. These pathways are tightly embedded into each other and the consequences of such organization will be detailed later. The C2 compounds used as carbon source enter metabolism at the level of the EMCP, from which they feed the central pathways. *M. extorquens *possesses also the ability to oxidize 26 additional compounds [26]. These compounds include a significant number of sugars, mainly pentoses. The reconstruction data suggest that such capability is due to the occurrence of soluble sugar dehydrogenases able to oxidize a wide range of pentoses and other sugars [27]; however, no assimilation processes were identified from the genome annotation. The reconstructed network contains a potential pathway for the utilization of two sugars (glucose and gluconate) as carbon source, although *M. extorquens *is not known to grow on these compounds. The identified pathway includes the periplasmic oxidation of glucose into gluconate, which could be internalized and catabolized through the pentose phosphate pathway (PPP) or the Entner-Doudoroff (ED) pathway. The glycolytic pathways are quite well established (see below) but the relevant sugar transport systems have low annotation confidence scores. Further experimental investigations will be necessary to determine whether *M. extorquens *possesses or not the entire enzymatic repertoire for sugar utilization.

The detailed examination of the biosynthetic pathways included in the GS network indicated incomplete lipopolysaccharide (LPS) biosynthesis. The pathways for keto-deoxyoctulosonate and lipid A synthesis and assembly were found, but the genes encoding the enzymes classically involved in heptose biosynthesis and in sugar incorporation onto the lipid A were missing. These observations suggest the occurrence of an unusual LPS structure in *M. extorquens *AM1. Consistently, no heptose or galactose was detected from the hydrolysis of cell material (see above) though significant contents in rhamnose (8.9 ± 0.9%) and glucose (5.6 ± 0.5%) were found. The GS network also included the biosynthetic pathways for carotenoids and bacteriochlorophyll A. The biosynthetic pathway of bacteriochlorophyll was complete in addition with the presence of the enzymes of the photosystem I. Several degradation pathways are missing in the GS network of *M. extorquens*, including the degradation of amino acids - e.g. histidine, arginine, tyrosine - and nucleotides. This is in agreement with physiological data showing that the occurrence of these compounds in the cultivation medium did not result in detectable metabolic activity.

The GS metabolic reconstruction showed that *M. extorquens *possesses a respiratory chain with alternative systems for electron inputs and outputs (Additional file 6). A great variety of potential electron donors could be identified, including a significant number of soluble periplasmic dehydrogenases transferring electrons to cytochrome c, including methanol dehydrogenase, formaldehyde dehydrogenase, and the already-mentioned soluble sugar dehydrogenase. Three terminal oxidases were also present, including two ubiquinol oxidases and one cytochrome c oxidase, suggesting that *M. extorquens *can adapt an aerobic metabolism to different oxygen levels. Besides oxygen, nitrate might represent a potential alternative electron acceptor.

### Organization of central metabolic pathways

C1 assimilation ensures the conversion of the C1-unit into precursor metabolites and involves a high number of metabolic pathways like C1 pathways, serine cycle, EMCP, TCA cycle, gluconeogenesis, anaplerotic reactions (Figure 1). They are connected by overlapping metabolites and enzyme reactions. The most central processes are interconnected cycles (serine cycle, TCA cycle) or pathways (EMCP, anaplerotic reactions). The serine cycle shares common reactions with gluconeogenesis (enolase), with the EMCP (malyl-CoA ligase, malyl-CoA lyase), with the TCA cycle (malate dehydrogenase), and with amino acid metabolism (serine hydroxymethyltransferase). The EMCP shares also reactions with the polyhydroxybutyrate (PHB) biosynthesis (acetyl-CoA C-acetyltransferase, acetoacetyl-CoA reductase), the TCA cycle (succinate dehydrogenase, fumarase), and fatty acid degradation (hydroxybutyryl-CoA (HBCOA) dehydratase, hydroxybutyryl-CoA dehydrogenase). The overall picture of *M. extorquens *central metabolism that emerges from these observations is an unusual series of metabolic pathways and cycles that are tightly embedded one into each other and allow operating almost as an entity. The C3 (2PG, pyruvate and PEP) and C4 (OAA and malate) intermediates play a critical role in the overall network organization. They represent the branching-points of the three main central metabolic pathways, i.e. the serine cycle, the EMCP and the TCA cycle, and of anaplerotic processes. Hence, they can be generated by different metabolic routes [28]. Accordingly, seven different reactions allow the inter-conversion of the five compounds (Figure 1). These reactions include processes inter-converting i) C3 into C3 (enolase, PEP synthase, pyruvate kinase), ii) C4 into C4 (malate dehydrogenase), iii) C3 into C4 (PEP carboxylase (PEPCL)), and iv) C4 into C3 (PEP carboxykinase (PEPCK), malic enzyme). Moreover, the C3/C4 inter-conversions include either reversible reactions (2PG/PEP and OAA/malate inter-conversion) or irreversible but opposite reactions (PEP/pyruvate and PEP/OAA inter-conversions). The result is a dense sub-network of reactions that provides alternative pathways for the same conversion [28] and the occurrence of potential substrate cycles [29].

### Identification of the sub-network operating during methylotrophic growth

The functional structure of the metabolic network operating during pure methylotrophic growth conditions of *M. extorquens*, i.e. growth on methanol as sole source of carbon and energy, was established by determining the sub-network of the GS metabolic model that includes all the reactions operating on methanol, thereafter referred to as the 'methylotrophic network' (Additional file 1). The identification of the methylotrophic and non-methylotrophic reactions was based on both theoretical and experimental considerations, including i) physiological parameters ii) genetic and biochemical data, iii) omics data (transcriptomic, proteomic, metabolomics), and extensive refinement. The reactions were confronted against all above criteria and were included or excluded a based on multi-criterion consideration and their overall score (Additional file 7). This reduction step was also a pre-requisite for in silico analysis of the methylotrophic network. The size of the GS model was too big to apply Elementary Flux Mode (EFM) analysis, a powerful tool to analyze the functional properties of metabolic networks from their topology [30], due to computational limitation. The Additional file 1 shows the diagram of the reduction process which was carried out in the following manner:

#### i) Methylotrophic growth of *M. extorquens*

The determination of the methylotrophic network was performed here to account for conditions where *M. extorquens *cells were grown exponentially in a minimal medium containing methanol as the sole added source of carbon and energy, NH_4_Cl as nitrogen source, and mineral salts, as described in material and methods. Such growth conditions allowed three levels of reduction of the GS metabolic model. First, the processes (transport and biochemical reactions) associated with the utilization of compounds (e.g. carbon sources, nitrogen sources, etc) included in the GS network but not occurring in the medium were removed. Similarly, the pathways and transport systems associated with metabolic end-products included in the GS network but not detected experimentally during methylotrophic growth were removed. Quantitative ^1^H-NMR analysis of culture supernatants collected after methylotrophic growth indicated that very few by-products accumulated in the medium, and only at negligible levels, allowing the removal of 187 reactions from the GS model. It was also assumed that in exponentially-growing cells no biomass degradation occurred, resulting in a further simplification of the network by removing the biochemical pathways specifically involved in the degradation or salvage of macromolecular components. This simplification resulted in the removal of 213 biochemical reactions. Some reactions associated with macromolecule degradation could be potentially involved in other metabolic processes, such as cofactor biosynthesis or recycling of anabolic by-products. Some of these reactions - a total of 13 - appeared to be relevant for growth on methanol and were kept in the methylotrophic network.

#### ii) Genetic and biochemical data

Literature data were used to further substantiate the methylotrophic network. More particularly, the phenotypes of gene deletion mutants were used to support the reduction process. From currently available literature, a total of 47 genes were shown to be essential during growth on methanol. Some of these genes encode multifunctional enzymes, so that a total of 51 biochemical reactions were associated with the 47 essential genes. All monofunctional enzymes (42 reactions) were kept in the methylotrophic model. For multifunctional enzymes, it could not be determined at this stage which reaction(s) was (were) responsible for essentiality, and other considerations were applied before making a decision as regard to their inclusion or exclusion of the methylotrophic network. In total, 2 reactions were excluded from genetic data analysis.

The enzyme assays available in the literature were considered to confirm the presence of biochemical reactions during methylotrophic growth, as well as their differential activities upon non-methylotrophic condition. Additional biochemical information from in vitro assay of particular reaction like spontaneous reaction or biochemical information in other microorganisms was used to validate reaction occurrence. In total, 13 reactions were excluded from biochemical data analysis.

#### iii) Omics data

The next reduction step was based on the comparison of omics data - including transtriptomics [17,31], proteomics [31], and metabolomics data [17,32-35], collected for both methylotrophic-grown and non-methylotrophic grown *M. extorquens *cells [17]. The molecular components corresponding to each type of omic data - e.g. protein for proteomics data - were kept in the methylotrophic network when they were identified to occur in methanol-grown cells, and/or their content was significantly higher - at least twice higher - than in non-methanol-grown cells, i.e. succinate-grown cells. The score of components identified from transcriptomics (differential expression), proteomics (spectral counting, differential expression) and metabolomics (identification) were assigned to their corresponding biochemical reactions via the GPR association. Taken together, the omics data were involved in the confirmation of the occurrence of 175 reactions, and the exclusion of 296 reactions. The final methylotrophic network contained 717 reactions and 722 metabolites, associated with 706 genes (Table 1, Additional file 7), and included approximately two thirds of the components of the GS network. To validate the topology of the methylotrophic network, non methylotrophic reactions were constrained to zero in the stoichiometric model of *M. extorquens *metabolism, and the reduced model was used to simulate growth performance on methanol. FBA simulations showed that the network supports a theoretical maximal growth rate of 0.20 h^-1^, which is consistent with experimental values [36]. Moreover, this value was close to the maximal growth rate calculated with the GS network (0.21 h^-1^), indicating that no significant growth capacities were lost during the reduction of the GS network and furthermore suggesting that about one-third of the total metabolic potential of the bacterium is not required for growth on methanol.

### Dissimilation capabilities of the methylotrophic network

The capability of methylotrophs to use methanol as sole energy and carbon source relies on the occurrence of both dissimilatory and assimilatory pathways, which fulfill all energetic and biosynthetic requirements, respectively. The reconstruction of the metabolic network of *M. extorquens *gave the opportunity to analyze the (system-level) organization of the two types of processes in this model methylotroph. Elementary flux mode (EFM) analysis, a powerful tool to analyze the functionality of metabolic networks from their topology [30], and FBA simulations, were carried out. Dissimilation and assimilation processes were first analyzed separately.

Dissimilation processes were defined here as processes resulting in the net conversion of methanol into CO_2 _and allowing energy conservation. The main dissimilation route is known to be the stepwise oxidation of methanol to CO_2 _using dedicated C1 pathways [5,15]. This process involves the periplasmic oxidation of methanol into formaldehyde, which is further oxidized to CO_2 _in the cytoplasm (see Figure 1). In this process one cytochrome C and two nicotinamide adenine dinucleotide (phosphate) (NAD(P)H) are released, assuming the pyridine nucleotide dependent formate dehydrogenase (FDH) operates. This 'cytoplasmic' route can fulfill both adenosine triphosphate (ATP) and redox requirements at the same time. In case cytochrome C and NADH are reoxidized by the most effective oxidative phosphorylation mechanisms, a maximal yield of 5 ATP/methanol is predicted (Table 3). The additional potential routes for methanol dissimilation within the methylotrophic network could be detailed from the *in silico *investigations (Table 3). A periplasmic route of formaldehyde oxidation can be predicted in case methanol-dehydrogenase-like enzyme XoxF would act together with a periplasmic formate dehydrogenase [37]. Such a route would not generate NAD(P)H and hence could only fulfill ATP requirements, albeit with reduced maximal yield (3 ATP/methanol). The network contains also other potential alternative mechanisms for the complete oxidation of methanol in which multi-carbon compounds are first generated and then completely oxidized to CO_2_. These processes would result from the combined action of C1 assimilation pathways (enabling the formation of multi-carbon compounds) and of catabolic pathways where multi-carbon compounds are fully oxidized to CO_2 _(via the TCA cycle or other decarboxylation reactions). The latter processes are the main energy conservation mechanisms upon utilization of C2 and other multi-carbon compounds, and are likely to be down-regulated during pure methylotrophic growth conditions. Nevertheless, the *in silico *analysis shows that the methylotrophic network contains the potential for these indirect dissimilation routes. They are however not efficient for energy conservation (Table 3) and are unlikely to operate upon methylotrophy from the energetic point of view.

**Table 3:** Dissimilatory processes in the methylotrophic network.

	max ATP	max NADH	max NADPH
Dissimilation processes	in mol.mol(methanol)-1		
MeOH - > CO2 (cytoplasmic FDH)	5	2	2
MeOH - > CO2 (periplasmic FDH)	3	0	0
MeOH + CO2 (Ser cycle) - > Acetyl-CoA - > TCA cycle - > 2 CO2	1	0.5	0.5
MeOH + n CO2 (Ser cycle + EMCP) - > other C2s, C3s, etc - > central pathways - > n+1 CO2	1	0.5	0.5

Types and number of dissimilatory EFMs detected in the methylotrophic network. For each type of dissimilation process, the theoretical maximum yields in ATP, NADH or NADPH are given.

### Assimilatory processes

The consequences of the particular organization of primary C1 assimilation in *M. extorquens *AM1 were analyzed by examining the processes allowing the conversion of methanol into each of the 13 key carbon precursors, including C1 (Me-THF), C2 (acetyl-CoA, glycine), C3 (L-serine, pyruvate, PEP, glyceraldehyde-3-phosphate), C4 (OAA, D-erythrose-4-phosphate (E4P), 3HBCOA), C5 (α-ketoglutarate, D-ribose-5-phosphate) and C6 (D-glucose-6-phosphate (G6P)). The number of EFMs ranged from 2018 to 6576 for the various carbon precursors (Table 4, Additional file 8). The serine cycle was involved in all assimilatory EFMs except for Me-THF, which can be also generated directly in the C1 pathways. This observation was consistent with the key role of the serine cycle pathway in methanol assimilation. The EMCP was involved in 93%, 95%, and 92% of the EFMs generating Me-THF, acetyl-CoA, and (R)-3-HBCOA, respectively. For all other carbon precursors, including the serine cycle intermediate glycine and L-serine, all assimilatory EFMs required the EMCP. These data emphasize the critical role of the EMCP (12 reactions), in addition to the C1 pathways (10 reactions) and the serine cycle (9 reactions), in methanol assimilation. Hence, the initial steps of C1 assimilation require the consecutive but obligatory operation of a high number of reactions. The minimal EFM length, representing the smallest number of reactions needed to convert methanol into each carbon precursor, was calculated for each of the 13 carbon precursors. The conversion of methanol into C3 compounds required at least 50 reactions, and the minimal number of reactions required to convert methanol into E4P was 63. Even for Me-THF and acetyl-CoA, the minimal EFM lengths were high (20 and 35, respectively). These data indicated that the primary assimilation processes, ensuring the conversion of methanol into carbon precursors, is a particularly complex process in *M. extorquens *AM1. Despite the complexity of methanol assimilation, the carbon precursors are produced from methanol with carbon yields that are similar to that observed on glucose for species like *E. coli *and *C. glutamicum*.

**Table 4:** Primary assimilation processes in the methylotrophic network.

compound/precursor biosynthesis	number of carbon in precursor	number of EFMs	Max. molar-Yield	Max. carbon-Yield	Minimal EFM length	EMCP utilisation
5, 10-methylenetetrahydrofolate (Me-THF)	1	2018	1.00	1.00	20	93%
acetyl-CoA	2	2440	0.45	0.91	35	95%
glycine	2	2054	0.42	0.84	62	100%
L-serine	3	2162	0.29	0.88	62	100%
D-glyceraldehyde-3-phosphate (GA3P)	3	2592	0.27	0.81	54	100%
phosphateenolpyruvate (PEP)	3	3390	0.32	0.97	53	100%
pyruvate (PYR)	3	3065	0.33	1.00	50	100%
oxaloacetate (OAA)	4	5366	0.32	1.29	51	100%
(R)-3-hydroxybutanoyl-CoA (3HBCOA)	4	2789	0.21	0.83	37	92%
D-erythrose-4-phosphate (E4P)	4	6576	0.20	0.81	63	100%
α-ketoglutarate	5	3806	0.20	1.02	54	100%
D-ribose-5-phosphate	5	4663	0.16	0.81	61	100%
D-glucose-6-phosphate	6	2592	0.14	0.68	58	100%

Number and properties of primary assimilatory EFMs detected in the methylotrophic network. The EFMs correspond to the processes allowing the conversion of methanol into 13 key carbon precursors

### Interdependencies of dissimilatory and assimilatory processes

**Figure 2:**
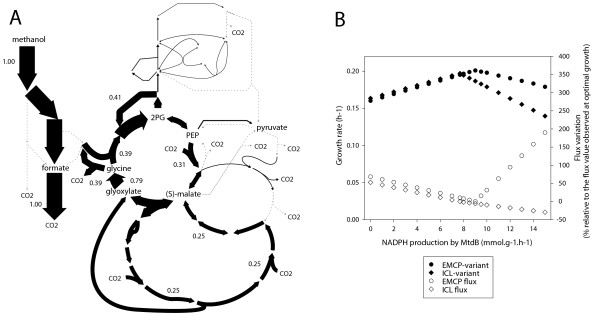
**Structural EMCP properties compared to ICL variant**. A. EFM (#122591) with the optimal biomass yield among the EFMs where biomass carbon is derived exclusively from CO_2_. B. FBA simulation of optimal growth rate depending upon a fixed proportion of NADPH/NADH produced by methylene- H_4_MPT dehydrogenases MtdA and MtdB in EMCP and ICL-variant.

**Figure 3:**
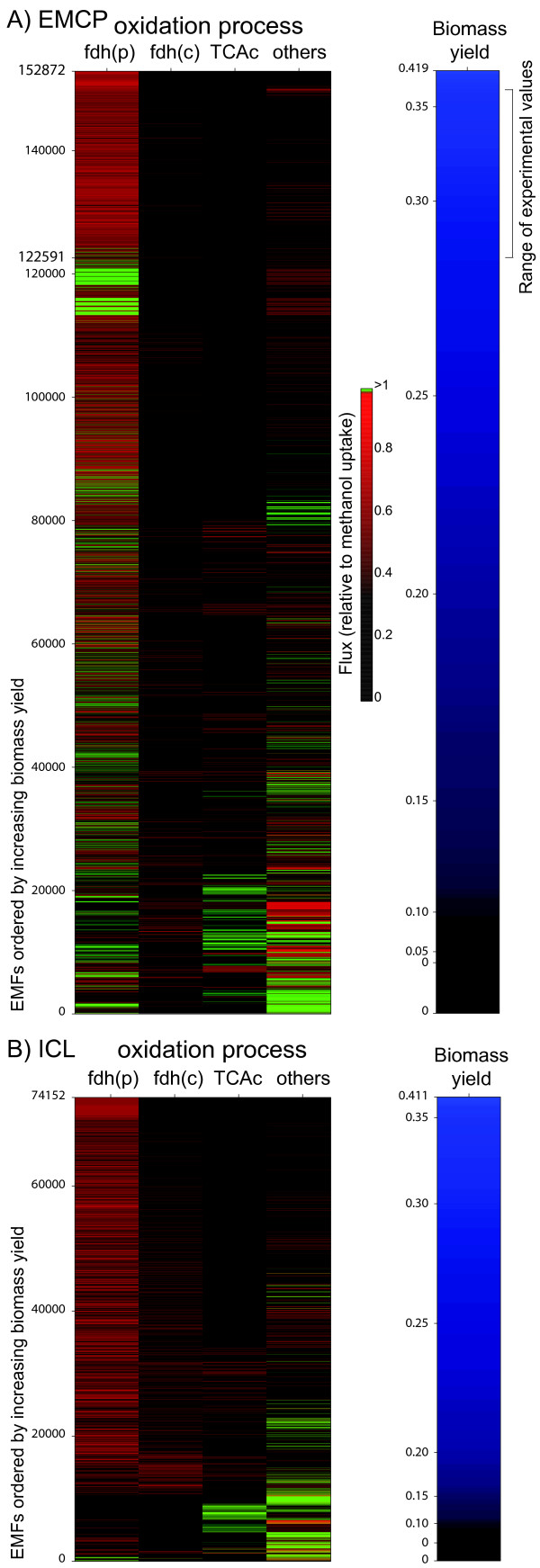
**EFM analysis of the balance between dissimilation and assimilation in EMCP and ICL variants**. The biomass-forming EFMs were calculated and sorted according to the biomass yield (from bottom to top, blue color scale). For each EFM, the flux through the main dissimilatory processes (see text for details) were extracted and plotted separately. Fluxes were expressed relative to the rate of methanol uptake, and were plotted using a colour-scale (black to green). First lane: cytoplasmic FDH, fdh(c); second lane: periplasmic FDH, fdh(p); third lane: TCA oxidation of acetyl-CoA, TCAc; fourth lane: other dissimilation process. A: EFMs calculated with the methylotrophic network, which contains the ethylmalonyl-CoA pathway (EMCP). B: EFMs calculated with the network variant where EMCP was replaced by the glyoxylate cycle (ICL variant).

The energetic efficiency of dissimilation processes determines the amount of energy available for assimilation and hence is a critical parameter of methylotrophic growth. The complete set of EFMs (152872) through the methylotrophic network was analyzed to investigate the relationships between dissimilation and assimilation processes. The EFMs were classified according to their biomass yields, and then to the various types of dissimilatory processes (Figure 2). In the EFM with the optimal biomass yield (0.42 g · g^-1^), 70% of methanol was directly oxidized via C1 pathways and the remaining was used for assimilation purposes. In a significant number of assimilatory EFMs, methanol was entirely oxidized to CO_2 _via the C1 pathways (Figure 2), meaning that no reduced carbon entered assimilatory pathways, and indicating that biomass could be fully generated from CO_2_. The highest biomass yield that could be obtained by such process was 0.283 g · g^-1 ^(EFM number 122591). In this EFM, (Figure 3A), carbon fixation is achieved by a process involving both the EMPC and the serine cycle. The process starts in the EMCP where two glyoxylate molecules are generated from one acetyl-CoA and two CO_2_. The two glyoxylate molecules enter the serine cycle to produce two glycine molecules. One glycine is converted by the glycine cleavage complex into one CO_2 _and one Me-THF. The latter compound allows the conversion of the second glycine molecule into serine, which is used in subsequent steps of the serine cycle, allowing both the regeneration of the initial acetyl-CoA molecule and enabling - through the operation of the entire mechanism - the formation of all carbon precursors needed for biosynthetic purposes. The overall carbon balance is 2 CO_2 _→ 1 glyoxylate. As this process requires the release of Me-THF via the glycine decarboxylase complex, it represents a distinct feature compared to the classical operation of the serine cycle. The carbon yield of the CO_2_-assimilation process is significantly lower than observed for methanol assimilation (40% vs 62%). The ATP needs are twice higher (7.2 vs 3.8 mol · mol(carbon assimilated)^-1^), and the redox needs are two to three times more elevated. Such high energetic costs can be covered by methanol oxidation, but the overall CO_2 _assimilation process is much less favorable than methanol assimilation. This CO_2 _assimilation process was not reported so far and is a direct consequence of the capability of the EMCP to ensure CO_2 _fixation.

### Substitution of the ethylmalonyl-CoA pathway by the glyoxylate cycle

The recently discovered EMCP is an alternative to the classical glyoxylate cycle for the biosynthesis of glyoxylate from acetyl-CoA in organisms lacking isocitrate lyase (ICL) [18,19,38]. To compare the metabolic properties conferred by the EMCP with that of the glyoxylate cycle, we generated a variant of the methylotrophic network lacking the EMCP but possessing the glyoxylate cycle. This was done by setting to zero the flux from crotonyl-CoA to propionyl-CoA and by adding the ICL reaction. Malate synthase, the enzyme of the glyoxylate cycle that catalyzes the condensation of acetyl-CoA and glyoxylate into malate, was not added since *M. extorquens *can use a combination of two enzymes to achieve the same reaction [39], as described also in *R. sphaeroids *[40]. As expected, the glyoxylate cycle was essential for methanol growth and was found in all assimilatory EFMs. The maximal biomass yield predicted for the ICL variant (0.41 g · g^-1^) was similar to that observed for the EMCP variant yield (0.42 g · g^-1^). To obtain such maximal growth, the rate of methanol oxidation in the ICL variant was smaller (61% vs 70%) and the NADPH requirements lower than in the EMCP variant, showing a higher energetic efficiency of the ICL variant. In contrast to the EMCP, the ICL variant was not found to allow entire biomass formation from CO_2_.

To investigate the potential role of the EMCP in redox balancing, we compared the capability of the two metabolic network variants to respond to varying NADPH production levels. FBA simulations were performed where the amount of NADPH produced by the Me-H_4_MPT dehydrogenase MtdB [41], which can use either NADH or NADPH, was varied from 0 to 15.0 mmol · g^-1 ^· h^-1 ^(0 to 100% of MtdB flux). For both the EMCP and ICL variants, the absence of NADPH production in the C1 pathways can be compensated by other NADPH-production systems, the PPP or malic enzyme. The theoretical maximal growth rate increased with NADPH production until a maximum is reached, which represents the optimal balance between NADPH production and growth. Maximal growth for the EMCP variant was obtained at higher NADPH production level than the ICL variant (9.0 vs 7.7 mmol · g^-1 ^· h^-1^), in agreement with the higher redox demand identified previously. The EMCP variant was able to maintain higher growth rates when the NADPH production was further increased. This capability was correlated with a significant increase of the EMCP flux. The increase in the EMCP flux was accompanied by the truncation of the serine cycle. Rather than being converted to OAA, PEP is converted into pyruvate via pyruvate kinase, which is further converted by pyruvate dehydrogenase into acetyl-CoA, which enters the EMCP. This pathway generates ATP (via pyruvate kinase) and releases NADH (via pyruvate dehydrogenase), resulting in a transhydrogenase-like mechanism where the redox equivalents are transferred from NADPH to NAD^+ ^in addition to the transfer to CO_2_.

### Fragility of the methylotrophic network

**Figure 4:**
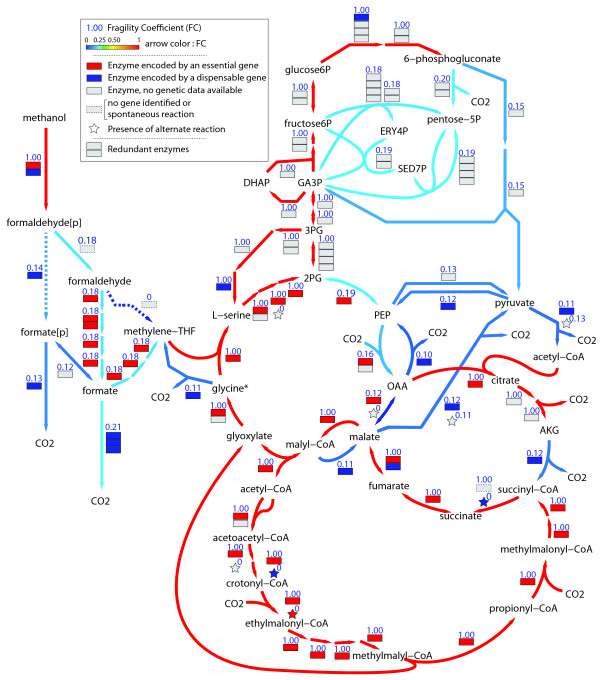
**Structural fragility of the methylotrophic network**. Prediction of reaction essentiality from Minimal Cut Set (MCS) analysis and comparison with experimental mutant phenotypes. The fragility coefficient (FC) calculated from MCS analysis is given for each reaction of *M. extorquens *central metabolism, and ranges from 0 (fully dispensable reaction) to 1 (essential reaction). Boxes next to reactions represent enzymes. Where available, the phenotype of the mutant lacking the gene encoding a particular enzyme is given by a color code (see legend). The occurrence of alternate reactions that are not displayed on the map is indicated by a star.

Robustness is an inherent property of a metabolic network and is defined as the capability of this network to operate despite one - or more - reactions are removed. The robustness of the methylotrophic network was analysed using minimal cut sets (MCSs), which correspond to minimal combinations - singlets, pairs, triplets, etc - of reactions whose removal blocks the operation of a target metabolic function [42]. The identification of all MCSs in a metabolic network allows the calculations of the fragility coefficient (FC) of each reaction. The FC of a reaction represents the probability that the metabolic system fails to achieve the target function when the reaction is removed. This approach was applied to analyze the robustness of the methylotrophic network using growth as the target metabolic function. A significant number of reactions (391) were found to have a FC of 1 and are therefore predicted to be essential (Additional file 9). Among these 391 reactions, 30% are catalyzed by multiple enzymes, indicating that enzyme redundancy is significantly used to avoid metabolic resilience in *M. extorquens *AM1. Most of the essential reactions (279) were found in biosynthetic pathways, which is consistent with studies performed with other organisms. The FCs of reactions found in central metabolism spanned over a wide range of values but distributed heterogeneously among metabolic pathways (Figure 4). Some processes, such as the C1- and carbohydrate pathways, and C3/C4 interconversion reactions, had low FCs and hence were predicted to be robust parts of the metabolism. Most other parts of the central metabolism had high FCs and hence were predicted to be fragile. Of the 84 reactions of the central carbon metabolism, 40 were found to be essential. The essential reactions concentrated in the primary assimilation processes (Figure 4). Most reactions of the serine cycle (67%) and of the EMCP (100%) were predicted to be essential for methylotrophic growth, indicating the assimilation processes to be highly fragile. The same observation holds true for gluconeogenesis. Because a particular reaction can be catalyzed by one or several enzymes, the essentiality of a reaction does not necessarily mean that the removal - by gene deletion - of one particular enzyme will be lethal. Among the 40 essential reactions found in central pathways, 29 were catalyzed by a single enzyme. Accordingly, 19 of these genes have been studied experimentally, and 95% of them were shown to be lethal for methylotrophic growth [43] (Additional file 10). For an essential reaction with multiple enzymes, one isoenzyme can compensate the lack of the other one(s). Genes encoding isoenzymes are therefore predicted to be not essential. However, mutant analysis showed that 4 out of 11 essential reactions with multiple enzymes found in primary assimilation processes, were encoded by gene where deletion were lethal for growth on methanol. This observation suggests that the products of these genes play an essential role during growth on methanol that cannot be compensated by the other potential enzymes catalysing the same reactions, or that they have different regulations, or both. Taken together, these data showed that the main processes of methanol assimilation are highly fragile in *M. extorquens *AM1. The robustness of the metabolism of *M. extorquens *AM1 was also calculated for a C2 compound (acetate), and for a C4 compound (succinate). The data were compared to that calculated for *E. coli *using the model of Klamt et al. [42,44]. The utilization of C2 compounds like acetate relies on the EMC pathway in *M. extorquens *and on the glyoxylate cycle in *E. coli*. Interestingly, the metabolic robustness of the central metabolism upon acetate growth was calculated to be lower in the former organism compared to the latter. Upon growth on succinate, which does not rely on C1-associated pathways but on common pathways (e.g. TCA cycle) that are similar in the two organisms, metabolic robustness was calculated to be similar between the two organisms. These observations suggest that metabolic fragility in *M. extorquens *is mainly related to the nature and organization of C1 (& C2) pathways in this organism.

### Distribution of metabolic fluxes during methylotrophic growth

**Figure 5:**
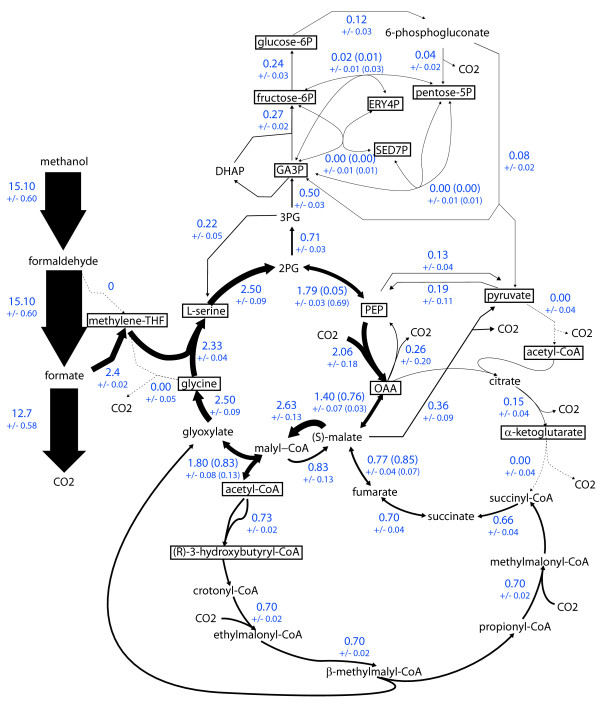
**Distribution of fluxes in the central metabolic network of *M. extorquens *AM1 upon methylotrophic growth**. Carbon fluxes were calculated from 183 isotopomer measurements (NMR + MS data) collected during steady-state growth of *M. extorquens *AM1 on [^13^C]-methanol. Fluxes are given in mmol · g^-1 ^· h^-1^, with standard deviations given below flux values. Exchange fluxes through reversible reactions are given within brackets. The width of the arrows is proportional to the flux value.

The distribution of metabolic fluxes during methylotrophic growth was determined using ^13^C-metabolic flux analysis (^13^C-MFA). ^13^C-MFA was already successfully applied to *M. extorquens *AM1, which provided valuable insights into the operation of central pathways [8]. The novel insights such as the operation of the EMCP [19], the organization of central assimilatory pathways [17], and the biomass composition (this study) necessitates the reinvestigation of metabolic flux analysis in *M. extorquens *AM1 during growth on methanol. To this end, a series of ^13^C-methanol labeling experiments was performed, and the isotopic information was monitored by both mass spectrometry and two dimensional nuclear magnetic resonance (2D-NMR) [45] (Additional file 11, 12). Such analytical combination provides critical information for the resolution of central pathways [19]. The flux distribution obtained from these investigations is displayed in Figure 5 and listed in Additional file 13, and the fitting accuracy and sensitivity analysis are listed in Additional files 12, 13, 14 and 15. The flux data obtained in the present were significantly different from that previously published [8]. This is mainly due to the major advances made in the meantime in the description and understanding of *M. extorquens *metabolism, and it is now known that the size and topology - and hence the possible carbon flows - of central carbon metabolism are more complete and much different than previously assumed. For this reason they can be hardly compared.

The flux data of Figure 5 indicated that 12.7 ± 0.6 mmol · g^-1 ^· h^-1 ^of methanol, 84% of methanol consumed (15.10 ± 0.60 mmol · g^-1 ^· h^-1^), was directly oxidized to CO_2 _within the C1 pathways. A release of CO_2 _was also observed within biosynthetic pathways (2.6% of consumed methanol) and central metabolism (5.4%). The release of CO_2 _in central metabolism was due to substantial fluxes through malic enzyme (0.36 mmol · g^-1 ^· h^-1^) and PEPCK (0.26 mmol · g^-1 ^· h^-1^), but was not associated with dissimilation processes. Indeed, the TCA contributed to only 1.1% of total CO_2 _release and operated in an incomplete and anabolic mode. The flux of C1 assimilation via Me-THF was 2.4 ± 0.02 mmol · g^-1 ^· h^-1^, which represents 16% of methanol uptake. The flux data clearly showed the central role of the serine cycle in distributing the carbon flow throughout the entire metabolic network to fulfill the requirements in carbon precursors. About 20% (0.5 mmol · g^-1 ^· h^-1^) was directed towards gluconeogenesis and carbohydrate pathways, and 30% was routed to the formation of pyruvate and TCA cycle intermediates. The release of acetyl-CoA by the serine cycle was significant (1.80 mmol · g^-1 ^· h^-1^). The major part (1.4 mmol · g^-1 ^· h^-1^) was recycled back to the serine cycle by the EMCP, and the remaining was used for anabolic purposes. The fixation of CO_2 _occurring within central pathways was calculated from the difference between CO_2_-utilizing and CO_2_-releasing fluxes, and was 2.4 mmol · g^-1 ^· h^-1^. This value was similar to the rate of methanol assimilation via Me-THF (2.4 mmol · g^-1 ^· h^-1^). These data were consistent with the observation that 50% of the biomass carbon derived from CO_2 _[7,46]. Such carbon balance can be obtained in case two molecules of glyoxylate are generated per turn of the EMCP [19], which requires that the propionyl-CoA produced in this pathway is not directly used for anabolic purposes but converted into glyoxylate. Accordingly, the replenishment of the glyoxylate pool from propionyl-CoA was almost identical to the direct release of glyoxylate within the EMCP (0.70 ± 0.02 mmol · g^-1 ^· h^-1^). No flux was found through the glycine cleavage complex, indicating that the CO_2 _assimilation mechanism identified from EFM analysis was not operating during pure methylotrophy, for chosen cultivation conditions. Surprisingly, a glycolytic flux through the Entner-Doudoroff pathway was observed. This flux (0.08 ± 0.02 mmol · g^-1 ^· h^-1^) was low compared to the rate of formate assimilation but was significant to fit the labeling data and represented about 14% of total pyruvate synthesis. This observation indicated that some carbon atoms were recycled through gluconeogenesis and glycolysis during methylotrophic growth.

The flux data provided valuable information regarding the C3/C4 pathways. The synthesis of pyruvate, which is required for various anabolic purposes, was proposed earlier to proceed via the conversion of PEP into pyruvate, via pyruvate kinase [6]. The flux data showed that pyruvate was synthesized by three different routes, including pyruvate kinase, the ED pathway and malic enzyme. The main flux was carried out by malic enzyme (0.36 vs 0.13 mmol · g^-1 ^· h^-1^). Moreover PEP synthase, which catalyses the reaction opposite to pyruvate kinase, is active and its flux (0.13 mmol · g^-1 ^· h^-1^) is higher than that of the latter enzyme. This observation indicated the occurrence of a substrate cycle between PEP and pyruvate due to the parallel activity of pyruvate kinase and PEP synthase, and in which 68% of pyruvate is recycled. Three additional substrate cycles were observed in this part of the metabolism. Two of them were related to C3/C4 inter-conversions: i) PEP/OAA cycling via PEPCL and PEPCK (13% of PEP recycled), and ii) PEP/malate/pyruvate cycling via PEPCL, malate dehydrogenase, malic enzyme and PEP synthase (4% of PEP recycled via malate and pyruvate). The fourth substrate cycle was observed between malate and (acetyl-CoA+glyoxylate). It relies on the reversible activity of the malyl-CoA lyase [47] and on the parallel operation of malate-CoA ligase and malyl-CoA thioesterase. Malate-CoA ligase is responsible for the release of glyoxylate and acetyl-CoA from malyl-CoA in the serine cycle. Malyl-CoA thioesterase catalyzes the opposite reaction. This enzyme is supposed to play a key role during growth on multicarbon compounds, but its activity is not expected during methylotrophic growth. Indeed, the enzyme activity is down regulated during growth in the presence of methanol [48]. However, a significant activity of this enzyme is still detected upon methanol growth [48], and represents 22% of the activity found on acetate. Such level of activity is likely to be sufficient to maintain a flux in the reaction, thereby resulting in substrate cycling. The flux data show not only that the latter cycle operates in *M. extorquens *AM1 during growth on methanol, but also that the extent of recycling is significant (32%). The energetic cost of metabolite recycling within the four above-mentioned processes was calculated from the flux data to be 1.3 mmol ATP · g^-1 ^· h^-1^, with the most expensive one (0.8 mmol ATP · g^-1 ^· h^-1^) being the malate/(acetyl-CoA+glyoxylate) cycle. The energetic cost was calculated to represent about 4% of the total production of ATP (29.3 ATP mmol · g^-1 ^· h^-1^). This energetic expense might represent a tradeoff between optimal metabolic efficiency and the ability to switch metabolic modes.

The total demand in NADPH during methylotrophic growth could be calculated from the fluxes in NADPH-utilizing reactions and biomass requirements, and was 5.6 mmol · g^-1 ^· h^-1^. The demand was mainly due for formate assimilation (2.3 mmol · g^-1 ^· h^-1^), biosynthetic requirements (1.8 mmol · g^-1 ^· h^-1^), and operation of the EMCP (1.4 mmol · g^-1 ^· h^-1^). The ^13^C-flux data showed also that the two NADPH-forming reactions found within central carbon pathways, i.e. isocitrate dehydrogenase and G6P dehydrogenase, contributed only negligibly (below 5%) to the total NADPH production. Hence, most of NADPH is generated alongside formaldehyde oxidation. From these data it can be calculated that the production of NADPH in the C1 pathway should be 5.3 mmol · g^-1 ^· h^-1 ^to close the NADP balance. To evaluate the ATP balance, flux variability analysis and FBA simulations were performed in which the methylotrophic network was constrained with the ^13^C-flux data, biomass requirements, experimental rates of growth and methanol uptake, and maintenance energy. Because the respiratory mechanisms by which the reduced cofactors (NADH, cytochrome C) generated in the C1 pathways are not clearly established in *M. extorquens *the ATP cannot be firmly established from the flux data. Nevertheless, the simulations showed that, if dissimilation proceeds via the cytoplasmic, NADH-dependent route at maximal ATP efficiency (Table 3), then the total production of ATP (44 mmol · g^-1 ^· h^-1^) would be in large excess compared to the requirements (29.3 mmol · g^-1 ^· h^-1^).

## Discussion

The genome scale metabolic network reconstructed in this work offers an integrated view of the current metabolic knowledge of the methylotrophic bacterium *M. extorquens *AM1. It provides new insights into the biochemistry of this organism and reveals the network-scale organization of metabolic processes as well as a first evaluation of the complete metabolic potential of this bacterium. The metabolic reconstruction allowed a detailed picture of the central carbon metabolism of the bacterium, which appears as a mosaic of common (TCA cycle, anaplerotic processes, gluconeogenesis, PPP, ED pathway) and specific (C1 pathways, serine cycle, EMCP) pathways, enabling growth on C1 and multicarbon (C2 to C4) compounds. The core of the central metabolism is organized as a highly unusual series of tightly embedded metabolic cycles that operate as an entity to achieve C1 assimilation during methylotrophic growth. The ability to assimilate C1 compounds relies on a complex metabolic machinery, in which the initial steps - from methanol to biomass precursors - require a particularly high number of reactions (e.g. at least 36 reactions to obtain acetyl-CoA). The entire process is strongly reductive and energy-consuming. Most of these reactions require enzymes and cofactors that are specific to C1 - or C2 - growth, and must be biosynthesized for the purpose of C1 or C2 utilization. Hence, the energetic costs for the biosynthesis and maintenance of this machinery are likely to be substantial for the bacteria. In addition, the network-scale analysis reveals that C1 assimilation is structurally fragile. Similarly to other metabolically specialized microorganisms [49], the core metabolism of *M. extorquens *is characterized by a high fraction of reactions that are essential for methylotrophic growth (almost 50%). In such networks robustness arise usually from enzyme (and genetic) redundancy, where multiple isoenzymes can catalyze essential reactions [49,50]. The genetic or biochemical deficiency in one isoenzyme can be compensated by another one. Accordingly, the percentage of multiple genes encoding essential reactions in specialized microorganisms is much higher than in generalist - or flexible - organisms such as *E. coli, B. subtilis *or *S. cerevisiae *(> 30% vs 10% redundancy, respectively) [50]. The high degree of redundancy (28%) observed in *M. extorquens *confirms the specialized metabolism of this methylotroph. Furthermore, among the essential reactions with multiple genes, a significant number of particular genes were shown experimentally to be essential for growth on methanol, suggesting that the alternative gene(s) have different functions or regulations, hence are not functionally redundant. Therefore, the number of genes essential for methylotrophy is high, and the risk that a gene mutation results in loss of methylotrophic capacity is elevated. This could explain, in part, the successes in the identification of such genes in the last 2 decades [43]. The observations emerging from metabolic network analysis are highly consistent with experimental evolution experiments in which a significant number of clones collected after prolonged cultivation (1500 generations) on succinate lost their methylotrophic capacity [36]. Taken together, available data suggest that a selection pressure is required to maintain methylotrophy in *M. extorquens*, indicating that the bacterium encounters frequently methanol in its natural environment and that its usage provides critical advantage in terms of ecological competitiveness.

The metabolic reconstruction data indicate also that a dense network of C3/C4 inter-conversions plays a critical role as a branch-point connecting specific and common pathways. Indeed, seven reactions interconnect three C3 (2PG, PEP, pyruvate) and two C4 (malate, OAA) intermediates, thereby strongly embedding the serine cycle, the TCA cycle, anaplerotic processes, and gluconeogenesis (via 2PG). Such topology allows a wide range of alternative metabolic routes and provides metabolic flexibility. Van Dien *et al. *[6,28] have emphasized the role of these processes in the metabolism of multi-carbon compounds. During growth on C4 compounds such as succinate, a functional TCA cycle is required and the generation of acetyl-CoA is ensured by pyruvate dehydrogenase. Hence, the conversion of C4 compounds into pyruvate is critical and can be achieved by redundant routes. During methylotrophic growth - in which a complete TCA does not operate -, the C3/C4 inter-conversions primarily ensure the opposite conversion of C3 intermediates into C4 intermediates in the serine cycle. They provide also alternative metabolic routes for such conversions, as observed with the significant conversion of PEP into pyruvate via PEPCL, malate dehydrogenase, and malic enzyme, though the role of this pathway is still unclear. The most striking feature is, however, the occurrence of substantial substrate cycling within the C3/C4 inter-conversions upon growth on methanol. In addition, substrate cycling was also observed between C4 (malate) and C2 (glyoxylate + acetyl-CoA), at the branch-point between the serine cycle, the TCA cycle, the EMCP and anaplerosis. Substrate cycles are resulting from the simultaneous operation of - non reversible - opposite reactions or processes, at the expense of energy. They can represent adaptation mechanisms allowing fast switching of metabolic processes [17,29]. The nature of the substrates cycles observed predicted during methylotrophic growth indicated that the entire set of reactions starting from PEP or pyruvate to acetyl-CoA and glyoxylate are operating as a fully reversible process. As mentioned above, these processes are the branching-point of the specific - i.e. serine cycle, EMCP - and common - i.e. TCA cycle, anaplerotic processes, and gluconeogenesis - pathways. They are also the starting point of a large number of biosynthetic routes, and the entry point of the utilization pathways of all C1, C2 and C3/C4 carbon sources used by the bacterium. Taken together, these data suggest that upon pure methylotrophic growth the occurrence of substrate cycling provides flexibility between specific and common pathways, thereby allowing fast switching of the metabolism from methanol to alternative carbon substrates.

Though *M. extorquens *AM1 is considered to be a methylotroph but not an autotroph, the *in silico *investigations revealed a potential of autotrophy in this bacterium, which relies on the unique property of the EMCP to enable CO_2 _fixation. The CO_2_-fixation mode involves a cyclic operation of both the EMCP and serine cycle to generate one glyoxylate from two CO_2_. It can potentially operate independently of methanol assimilation in *M. extorquens*, but was not observed during methylotrophic growth in our investigations. Because the genome of *M. extorquens *contains the complete information for a photosynthetic machinery, it is tempting to speculate that this bacterium may operate in a photoautotrophic mode. However, there is currently no experimental evidence of such behavior in *M. extorquens*. The question of the role, benefit and conservation of this pathway in *M. extorquens *and other organisms is still unclear. The EMCP is more complex and energy-demanding than the glyoxylate cycle though it provides a higher carbon balance for assimilation. In photosynthetic methanol utilizers, carbon dioxide functions as an electron sink for the excess electrons in methanol [51]. It was recently proposed that CO_2 _fixation could represent an alternative mechanism of cofactor recycling in bacteria [52]. The potential role of the EMCP in such mechanism was recently shown from investigations of CBB mutants defective for the reductive PPP pathway in acetate-grown *R. sphaeroides *[53]. Our investigations show that the EMCP can potentially play such a role in *M. extorquens *upon methylotrophic growth. If further investigations are required to determine the actual physiological benefits of the EMCP in serine cycle methylotrophs, our investigations show that this pathway can potentially play the role of a redox-balancing mechanism or of an autotrophic pathway.

## Conclusions

The unusual organization of the central carbon metabolism of *M. extorquens *AM1 allows efficient utilization of C1 compounds via highly specific - and fragile - pathways but is versatile enough around a flexible backbone of C2/C3/C4 inter-conversions to allow switching to other carbon sources. These observations showed that the bacterium maintains active metabolic processes that are not needed for methanol utilization but allow adaptation to other carbon sources. This hypothesis is consistent with the observation that methanol is produced by plant with methanol release in the morning [54,55]. This work emphasizes that the metabolism of the bacterium is adapted to its lifestyle not only in terms of enzymatic equipment, but also in terms of network-level structure and regulation. It suggests that the metabolism of the bacterium is adapted both structurally and functionally to an efficient but transitory utilization of methanol. This work illustrates that the combination of GS network modeling and experimental approaches provides novel insights into the biochemistry and physiology of methylotrophic bacteria, which could be extended to obligate methylotrophs and to the comparison of serine cycle versus RuMP- and CBB-utilizing methylotrophs. Methanol is currently regarded as a highly attractive raw material for microbial bioprocesses [56]. Comprehensive, system-level understanding of methylotrophic metabolism is also expected to improve the biotechnological value of methylotrophy, and this knowledge will serve as a sound basis for a rational remodeling of existing biosynthesis routes and for the design of new synthetic pathways [1].

## Methods

### Network reconstruction

The genome-scale (GS) metabolic network of *M. extorquens *AM1 was reconstructed using procedures recommended for the generation of high-quality reconstructions [21]. The detailed procedure is given in Additional file 1. Briefly, the process of metabolic reconstruction included the following steps:

1. Generating a draft reconstruction. The genome information was extracted from the MicroScope database [57] on December 9^th^, 2009. Genes annotated for metabolic functions were selected and assembled with biochemical information collected from literature. The data were completed using metabolic databases - mainly Metacyc [24], KEGG [22] -. During the reconstruction process, systematic Blast and interrogation of metabolic databases were performed to refine weak - or missing - genome annotation information or new published data. In some cases this process leads to the re-annotation of genes and the publicly available annotation was corrected accordingly.

2. The reconstruction was refined from all genetic, biochemical, and physiological data available for *M. extorquens *and related species, metabolic (Metacyc, KEGG) and transporter (TCDB, http://www.tcdb.org/) databases, and from self-expertise on methylotrophy (Additional file 1). Metabolite information such as the name, molecular formula and metabolic database identifiers of compounds were included in the network, and refined from available metabolomics data for 157 metabolites [35]. Neutral formulas obtained from PubChem http://pubchem.ncbi.nlm.nih.gov/ were used to validate reaction stoichiometry (mass balancing) including proton balance.

3. Generation of a Gene-to-Protein-to-Reaction (GPR) association network. The GPR association was designed to describe explicitly all the relationships between molecular species and functional activities. Specific identifiers were assigned to enzymes, reactions and metabolites.

4. Gap-filling. A draft metabolic map, drawn using the software Cytoscape [58], was used as starting point for the gap filling process. Gaps were identified from stand-alone reactions or metabolites, and from missing connections in essential metabolic processes. Spontaneous reactions, and reactions or transports without associated genes in *M. extorquens *genome, were added according to metabolic (Metacyc, KEGG) and transport (TCDB) databases.

5. Conversion of the reconstruction into computational format. The reconstruction was loaded into the software CellNetAnalyser [25]. The consistency of the reconstructed network was evaluated from *in silico *investigations (modeling) and from the ability of the network to explain growth on the most studied carbon sources, namely methanol and succinate. Experimental information [26] was used to identify substrate utilization. Exchange reactions - i.e. exchange with environment - were finally added corresponding to known substrates usage and minimal medium composition.

6. Quality assessment. The quality of the reconstructed network was determined according to [21] by assigning a confidence score to each individual reaction, depending on the evidence for the presence of the reaction, with the highest score given to experimentally demonstrated reactions and the lowest score given to gap-filling reactions.

The detailed list of reactions, metabolites, and other network components, and the GPR association network are given in Additional file 2, 3. The computer model of iRP911 written in Systems Biology Markup Language (sbml) is given in the additional file 16.

### Determination of the chemical composition of cells

*M. extorquens *AM1 was grown in fed-batch mode in mineral medium containing methanol as sole carbon and energy source, as described in [19]. For cell dry weight (CDW) determination, 30 ml of culture were centrifuged in a 50 ml falcon tube and washed with de-ionized water and dried to constant weight at 80°C. Falcon tubes were incubated for several days at 80°C prior to use. For other measurements, cells were harvested by centrifugation at 5000 g during 5 min. Cell pellets were frozen in liquid nitrogen and stored at -20°C until analysis.

#### i) Lipid content

whole cell hydrolysis with subsequent acid methylation of fatty acids was carried out as described in [38] with slight modifications. Cells (10-20 mg CDW) were hydrolyzed with 4 ml of 15% NaOH (w/v) in methanol/water (1:1, v/v) for 30 min at 100°C. Fatty acid methyl esters (FAMEs) were obtained by addition of 8 ml 6 M HCl/methanol (13:11, v/v) and incubation for 2.5 hrs at 80°C. An internal fatty acid standard (3 mg C15:0) was added before hydrolysis for quantification purpose. The methylation yield was measured from the addition of a FAME standard (3 mg C19:0 FAME) after the methylation step. FAMEs were extracted with 5 ml hexane/methyl-tert-butyl ether (1:1, v/v) and washed with 6 ml 1% NaOH in water (w/v). Extracted FAMEs were analyzed by gas chromatography - flame ionization detector (GC-FID) (Agilent Technologies 6850 with 7683B Series injector and FID detector) and a HP-5 column, length 30 m, I.D. 0.25 mm, film 0.25 μm (Agilent Technologies). Helium was the carrier gas with a column flow of 2.4 ml/min; detector temperature was set to 300°C, and inlet to 250°C. A temperature gradient was run from 190°C to 260°C at 5°C per min. A sample volume of 1 μl was injected with a spilt ratio of 30.

#### ii) Protein content

total proteins were quantified by the Biuret method [59], using bovine serum albumine (2 mg/ml) as standard. This method is independent of protein composition [60]. Cells were hydrolyzed in 0.75 ml 1N NaOH (1-2 mg/ml CDW) at 100°C for 5 min. After addition of 0.25 ml of 2.5% CuSO_4 _(w/v), samples were centrifuged and absorption was measured at 550 nm. The composition in amino acids of proteins was determined following hydrolysis in 6 M HCl at 110°C for 22 hours under argon samples were dried and derivatized using the AccQ-Tag™ Ultra derivatization chemistry (Waters Corp., Milford, MA, USA) according to the manufacturer's instruction. Amino acid derivatives were separated by UPLC (Waters Corp., Milford, MA, USA) using the AccQ-Tag™ Ultra standard hydrolysate conditions. Amino acid derivatives were detected by UV absorbance.

#### iii) Carbohydrate content

the carbohydrate content was measured after hydrolysis of the entire cell pellet. A two-step derivatization was used to convert carbohydrates into oxime trimethylsilyl derivates [61], which were analyzed by GC-FID. For glucose and rhamnose quantification, cells (1-3 mg CDW) were directly subjected to 200 μl 2 M HCl at 80°C for 4 hrs or to 4 M HCl for 16 hrs for glucosamine quantification, respectively; carbohydrates were stable under these conditions. After neutralization, 50 μl of 25 mM lactose solution was added as an internal standard. Samples were vacuum-dried and derivatized for 40 min with 150 μl 0.5 M hydroxylamine·HCl in pyridine at 80°C. After addition of 110 μl (trimethylsilyl)trifluoroacetamid (BSTFA), samples were incubated for another 20 min. Separation and quantification of the derivatives were performed by GC-FID as described under lipids except that column flow was set to 2.7 ml/min and temperature gradient was run from 160°C to 310°C with 7°C per min.

#### iv) Polyhydroxybutyrate (PHB) content

The measurement of the PHB content was performed according to [62,63]. Cell pellets (3-4 mg) were lyophilized and subjected to acid methanolysis with 3% H_2_SO_4 _(v/v) in methanol/chloroform 1:1 (v/v) for 2.5 hrs at 100°C. Benzoic acid was added as an internal standard prior methanolysis. Methyl-hyroxybutyryl monomers were extracted after addition of water (20% v/v) and vigorous mixing, The organic phase was analyzed by GC-FID with a DB-WAX column, length 15 m, I.D. 0.32 mm, film 0.5 μm. Column flow was set to 1.8 ml/min, detector temperature to 270°C and inlet temperature to 240°C. A sample volume of 1 μl was injected with a split ratio of 2. Temperature gradient was run from 90°C to 230°C at 40°C/min.

#### v) DNA content

The DNA content was calculated from that in *E. coli *[64], using appropriate corrections to account for the size of *M. extorquens *genome and for its growth rate on methanol.

#### vi) RNA content

The RNA content was determined from the amount of ribose released after acidic hydrolysis (2M HCl for 2 hrs), assuming that all ribose was derived from RNA. The hydrolysis yield was determined from commercial RNA and data were corrected accordingly. Ribose was quantified as described under iii).

#### vii) Polyamine content

The polyamine content was calculated from that in *E. coli*. The occurrence of putrescine in methanol-grown *M. extorquens *cells was also confirmed by GC-MS-MS.

#### viii) Carotenoid content

Data were taken from [65]. Based on their experimentally determined chemical properties, they were assumed to be spirilloxanthin-like carotenoids.

#### ix) Content in intracellular metabolites

Data - which included both the nature and amounts of metabolites - were taken from [34,35], [32,33], [66], and [7]. The amounts of Coenzyme A thioesters were determined by P. Kiefer (unpublished data). The content in tetra-aminoptherin and related cofactors were obtained from [7,66]. The contents in other cofactors were calculated from that in *E.coli *[64], using appropriate corrections.

#### viii) Inorganic ions

The amounts of inorganic ions were calculated from that in *E.coli *[64], using appropriate corrections.

The complete details of the biomass composition of *M. extorquens*, as they result from the above investigations or calculations, are given in Additional file 4.

### Cultivation and labeling experiment

Batch cultivations (three biological replicates) of *M. extorquens *AM1 were carried out at 28°C in minimal medium, in a bioreactor (Infors-HT, Bottmingen, Switzerland), as described previously [19]. Cultivations carried out for the purpose of steady-state [^13^C]-methanol experiments were performed like in [19]. The cultivations were aerated with 5% natural labeled CO_2 _to remove the ^13^CO_2 _produced by the bacteria from [^13^C]-methanol. Under this condition, only 4.6 ± 0.4% of total CO_2 _was found to derive from [^13^C]-methanol oxidation.

### *In silico *calculations

The metabolic network - containing 1139 (m) reactions 977 (n) metabolites - was converted into a mathematical model corresponding to a m × n matrix defining the stoichiometric coefficient of reactions. Calculations of steady-state fluxes were performed using the software CellNetAnlyser [25] and Matlab (Mathworks, Inc.). Flux Balance Analysis (FBA) calculations were performed using various objective functions, as indicated in the text. Despite the genome annotation revealed the occurrence of a potential photosynthetic machinery, all calculations were performed assuming that no photosynthesis operated since no phototrophic behavior was reported for *M. extorquens*. Calculation of EFMs in the methylotrophic network was performed using the solver EFMTool [67]. They were calculated assuming no maintenance energy. To be compared with experimental data, the obtain biomass yields (Y) of the EFMs were calculated as following:

$$Y=\frac{\mu_{max}}{\frac{q_{si}\times \mu_{max}}{\mu_{i}}+NGAM_{s}}\times \frac{1000}{MW_{s}}$$

With μ_max_: theoretical maximum growth rate (calculated to be 0.201 for the methylotrophic network from FBA simulations); μ_i_: growth rate of the EFM_i _(= 0.1); q_si_: substrate uptake rate of the EFM_i_, in mmol · g^-1 ^· h^-1^; NGAM_s_: corresponding substrate uptake to fulfill non-growth associated maintenance energy, i.e. 1.9 mmol · g^-1 ^· h^-1^; MW_s_: molecular weight of the substrate. Minimal cut sets [42] were calculated on the calculated EFMs, using biomass production as target function.

### ^13^C Metabolic Flux Analysis

The distribution of metabolic fluxes during methylotrophic growth was determined from ^13^C-labeling data collected during steady-state growth of *M. extorquens *on [^13^C]-methanol. The distribution of ^13^C-isotopomers in metabolites can be accurately determined by NMR or MS [45,68,69], alone or in combination [19]. Here the ^13^C-isotopomers of proteinogenic amino-acids were measured by the two methods. NMR spectra were monitored as described in [19] and LC-MS analysis were performed using Rheos 2200 HPLC system (Flux Instruments) coupled to an LTQ Orbitrap mass spectrometer (Thermo Fisher Scientific), equipped with an electrospray ionization probe and the amino acid were separated on a pHILIC column (150 × 2.0 mm, particle size 5 μm; Sequant, Umea, Sweden), following a procedure described [34]. A total of 193 isotopomer data - including 137 NMR data plus 56 MS data - were collected (Additional file 12). The metabolic network considered for flux calculations contained 65 reactions - including 7 reversible reactions - describing *M. extorquens *central metabolism, according to the topology of the methylotrophic network (Additional file 17). Flux calculations were performed using the software 13C-Flux [70], which uses both mass balances and carbon atom transitions to describe the metabolic. The methanol uptake rate and the requirements in biomass precursors, determined from data in Additional file 4, 11, were constrained. The confidence on the measured fluxes was determined using the sensitivity analysis module of 13C-Flux. Results were expressed as absolute fluxes in mmol.g^-1^.h^-1 ^+/- standard deviations.

## Authors' contributions

RP performed the reconstruction and the modeling. KS and PK performed the determination of the biomass composition. RP performed the *in silico *analysis (EFM, MCS, FBA). RP and SM performed the NMR measurement. RP and PK performed the MS measurement. RP performed the data treatment. RP performed the metabolic flux analysis and statistical analysis. RP and JCP analyzed the results. RP, JV and JCP wrote the manuscript. RP, JV and JCP conceived the study. All authors read and approved the final manuscript.

## Supplementary Material

Additional file 1 **Work flow of the reconstruction and reduction processes**. The work flow of the reconstruction was performed similarly to the protocol recommended for the generation of high-quality reconstruction (Thiele & Palsson, 2010). Click here for file

Additional file 2 List of reactions of the reconstruction (iRP911) Click here for file

Additional file 3 List of metabolites of the reconstruction (iRP911) Click here for file

Additional file 4 Detailed biomass composition Click here for file

Additional file 5 **Table of substrate usage by *M. extorquens *AM1 from experimentally observed phenotype and Flux Balance Analysis using the genome scale network (iRP911)**. Click here for file

Additional file 6 **Electron flow through the metabolic network of *M. extorquens *AM1**. The schemas represent the reactions involved in electron flow in *M. extorquens *AM1like it appear from the network reconstruction (iRP911). Detail on the reaction, given with identifiers of the type R-XXXX, can be found in the Additional file 2. Click here for file

Additional file 7 **Methylotrophic network**. The reactions of the GS network were included or excluded from the methylotrophic network based on multi-criteria analysis. The list of considered criteria is given here as well as the score for each reaction of the GS network. The list of reactions included in the methylotrophic network are indicated as '1' in the 'methylotrophic network' column. Click here for file

Additional file 8 **EFM analysis of the primary assimilation pathways and connectivity of biomass precursors**. The EFMs were calculated for the conversion of methanol into 13 key carbon precursors. The main properties of the calculated EFMs are given. Click here for file

Additional file 9 **Reaction essentiality in the methylotrophic network**. The graph displays the essentiality or dispensability of reactions and showx the experimental evidence for gene essentiality. Reaction essentiality was analyzed using Minimal Cut Set calculation [42] applied to the set of EFMs [30] allowing biomass production from methanol. Fragility Coefficients (FCs) were calculated from the MCSs [42]. Reactions having a FC of 1 were identified as essential (red arrows), and reactions with a FC < 1 were considered as dispensable (blue arrows). The enzyme(s) catalyzing the network reactions are represented by boxes. The experimental phenotypes of mutants affected for these enzymes are displayed using a color code: red box: lethal phenotype, blue box: non-lethal phenotype, black box: no experimental data available. The accuracy of the model prediction is indicated upper the bar for each class of reaction. Click here for file

Additional file 10 Table of published mutant phenotypes and associated genes and reactions Click here for file

Additional file 11 Growth parameters measured for the 3 replicate 13C-methanol labeling experiments Click here for file

Additional file 12 Fitting of the isotopomers data collected during 13C-methanol labeling experiments Click here for file

Additional file 13 **Flux distributions and sensitivity analysis**. Only C1 assimilation was considered during flux calculation due to the high difference in range of C1dissimilation and assimilation. Measured methanol uptake rate was considered subsequently. Click here for file

Additional file 14 **Quality of isotopomer fitting**. Comparison of experimental and collected isotopomer values for the three biological replicates. The isotopomer data include both LC-MS and 2D-NMR (HSQC and TOCSY) data. Flux calculation and fitting were performed using the software 13CFlux (Wiechert et al, 2001). A) Experimental values (+/- standard deviation) are plotted against theoretical values. B) Residuum of the calculated data. Click here for file

Additional file 15 **Flux variability in the 3 biological replicates**. Comparison of the flux distribution obtained for the three biological replicates. The flux calculation and the sensitivity analysis were performed using the software 13CFLUX (Wiechert et al, 2001). The fluxes were normalized by the flux of entry of the C1-units in central metabolism (SHMT: serine hydroxymethyltransferase). Flux distributions were found to be similar except slight changes through the C3/C4 interconversions (pyruvate kinase (PK), pyruvate dikynase (PPDK), malic enzyme (ME) and the phosphoenolpyruvate carboxykinase (PEPCK)), and through the Entner-Doudorof pathway. Click here for file

Additional file 16 **Computer model written in Systems Biology Markup Language of the genome-scale metabolic network iRP911**. Click here for file

Additional file 17 **Ftbl file describing the network used for flux calculation**. Click here for file
