# Supplementary material for: Genome-scale reconstruction and system level investigation of the metabolic network of Methylobacterium extorquens AM1
Source: BMC Syst Biol. 2011 Nov 10;5:189. doi: 10.1186/1752-0509-5-189 (PMC3227643; doi:10.1186/1752-0509-5-189)
Supplement: Additional file 1 — Work flow of the reconstruction and reduction processes. The work flow of the reconstruction was performed similarly to the protocol recommended for the generation of high-quality reconstruction (Thiele & Palsson, 2010). [file 1752-0509-5-189-S1.PDF]

WORK FLOW OF THE RECONSTRUCTION & REDUCTION PROCESSES

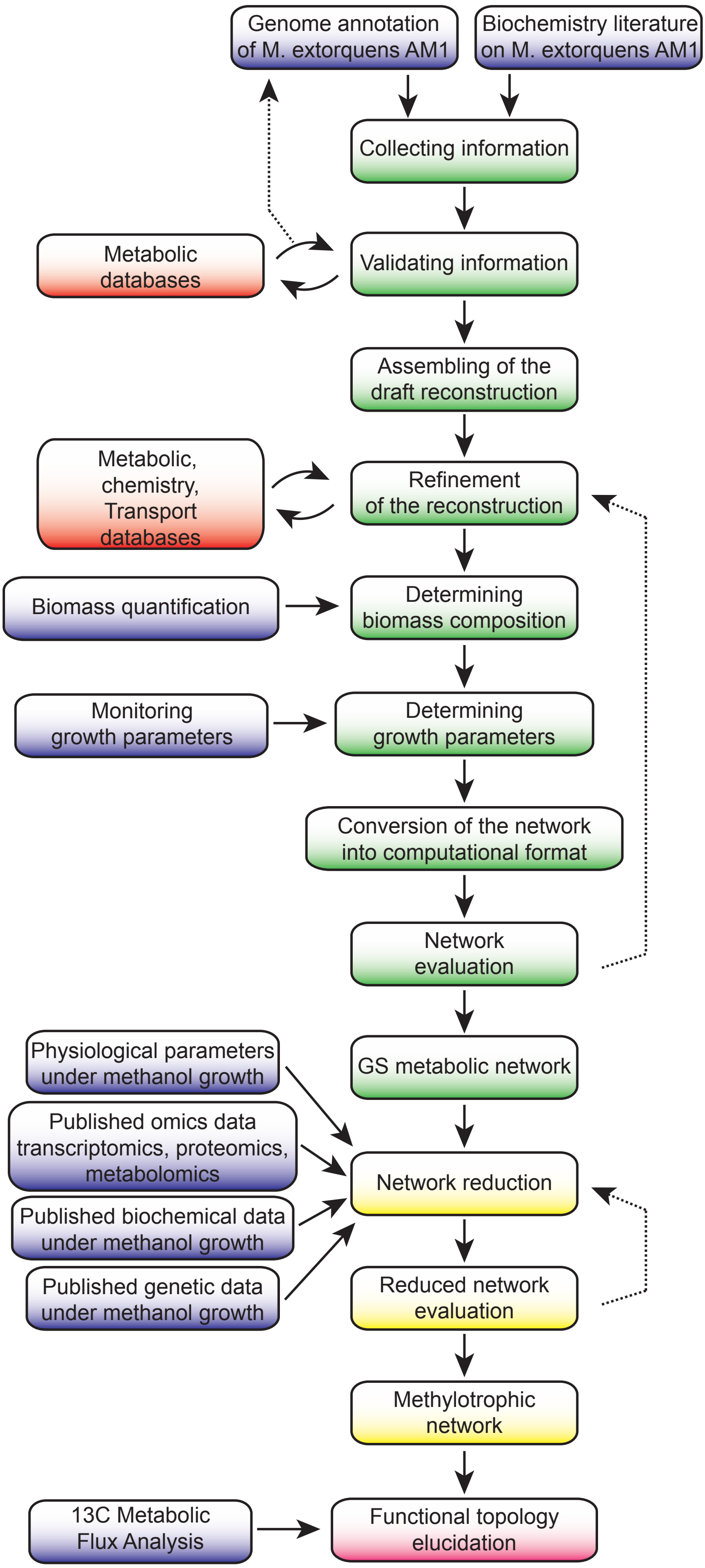

Collecting information
1. Download genome annotation
2. Selecting candidate assign to metabolic function
3. Extract available information :
   - genes identifier
   - genes name
   - genes product name
   - genes product localisation
   - metabolic function (from EC number or product name)
   - assigned metabolic pathway
   - confidence score of the annotation
4. Collect *M.extorquens* AM1 specific biochemystry information
   - catalysed reaction
   - cofactor usage
   - stereochemistry when avalaible
   - identified associated enzyme or gene

Validating information
5. refining metabolic function from metabolic reaction databases
6. refining genome annotation supported by blast query

Assembling of the draft reconstruction
7. Creating gene−protein−reaction association
8. extending localisation and pathway assigment to reaction

Refinement of the reconstruction
9. verification of substrate and cofactor usage
10. collecting of metabolite identifier and neutral formula
11. verification of reaction stoichiometry and mass balances
12. Determining reaction directionality
13. Verifying gene−protein−reaction association.
14. Verifying localisation and pathway assignment
15. Addition of spontaneous reactions to the reconstruction.
16. Addition of extracellular and periplasmic transport reactions.
17. Addition of exchange reactions.
18. Drawing of a draft metabolic map
19. Identifying gaps from standalone reactions in the metabolic map
20. Addition of gap reactions to the reconstruction.
21. Repeat steps 5 to 14 for all genes and reactions
22. Determine and add confidence score.
23. Addition of references and notes.

Determining biomass composition
24. Experimental identification and quantification of the chemical composition of the cell grown on methanol
25. Addition of biomass reactions.
26. Addition of ATP-maintenance reaction (ATPM).
27. Addition of demand reactions to the reconstruction

Determining growth parameters
28. Experimental monitoring of physiological parameters
29. Determining growth medium requirements.

Conversion of the reconstruction into computational format
30. Loading of the reconstruction into CellNetAnalyser
31. Setting of the biomass constituents and assembly routes.

Network evaluation
32. Identifying and addition of missing exchange reactions to model.
33. Check for blocked reactions and gap identification.
34. Addition of gap reactions accordingly to 33.
35. Setting of exchange constraints to 0.
36. Test for stoichiometrically balanced cycles.
37. Analyzing of the directionality of each reaction participating in a stoichiometrically balanced cycles
38. Adjusting the directionality for all reactions identified previously
39. Set exchange constraints corresponding to minimal medium and methanol as carbon sources
40. Addition of a demand function for each biomass precursor
41. Testing if the biomass precursors can be produced
42. Identifying missing reactions responsible for infeasability of the precursor biosynthesis
43. Addition of gap reactions accordingly to 42.
44. Testing of growth capability.
45. Testing of oxidation capability.
46. Addition of gap reactions accordingly to 44 and 45.
47. Repeat steps 5 to 14 for all added genes and reactions during gap filling steps.
48. Repeat network evaluation iteratively.

Reduction to the methylotrophic network
49. Identifying reactions involved and not invoved in methylotrophic growth from:
   - growth conditions and physiologycal parameters of M. extorquens AM1 growth on methanol
   - omics: transciptomics, proteomics, metabolomics
   - genetic and biochemical data
50. Setting to 0 flux throught reaction identified as not involved during methylotrophy

Reduced network evaluation
51. Testing if the biomass precursors can be produced
52. Identifying reactions responsible for infeasability of precursor biosynthesis
53. Re-evaluate reduction criterion for these reactions
54. Testing of growth capability using methanol as sole carbon and energy source.
55. Identifying reactions responsible for growth failure.
56. Re-evaluate reduction criterion for these reactions

Functional topology elucidation
57. 13C Metabolic Flux Analysis
