# Supplementary figures and images for: Genome-scale reconstruction and system level investigation of the metabolic network of Methylobacterium extorquens AM1
Source: BMC Syst Biol. 2011 Nov 10;5:189. doi: 10.1186/1752-0509-5-189 (PMC3227643; doi:10.1186/1752-0509-5-189)

# Electron flow in *M.extorquens* AM1 respiratory chain

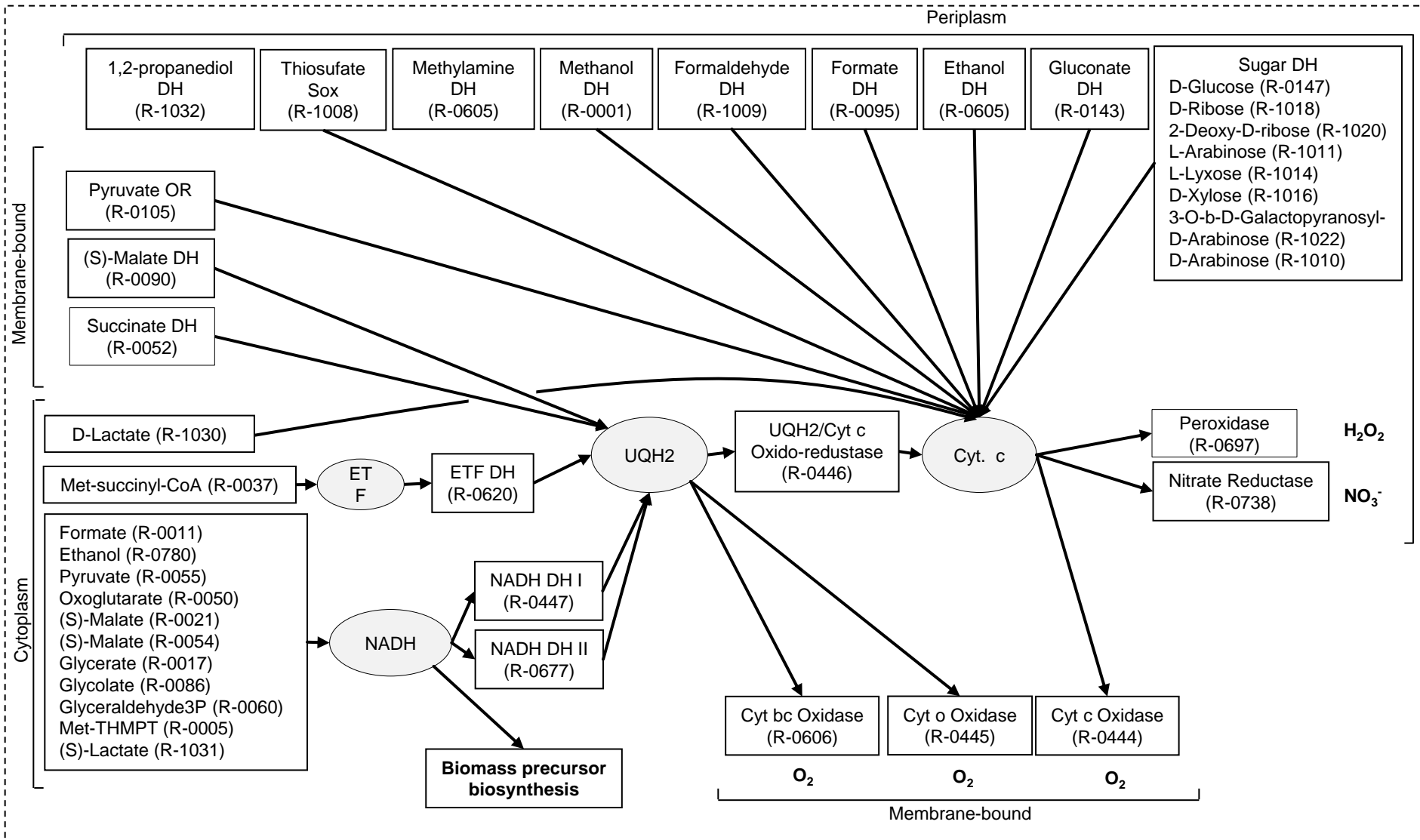

## Electron flow via NADPH in *M.extorquens* AM1

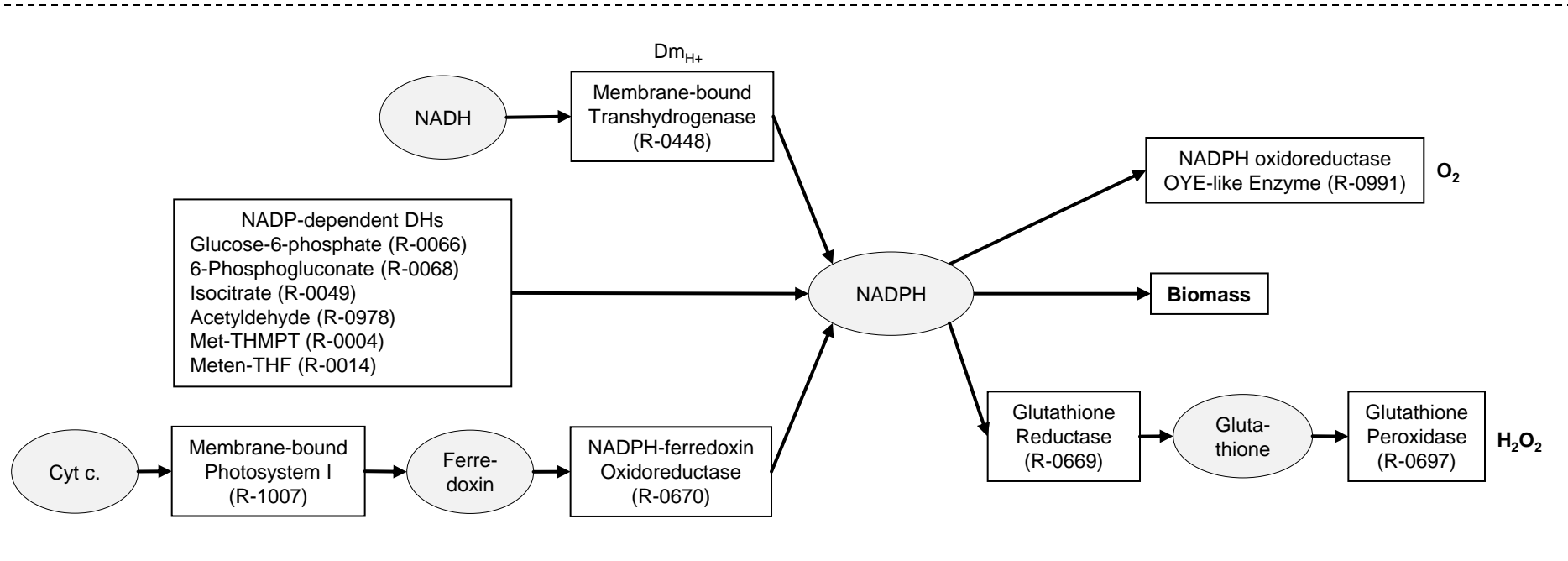

Supplement: Additional file 6 — Electron flow through the metabolic network of M. extorquens AM1. The schemas represent the reactions involved in electron flow in M. extorquens AM1like it appear from the network reconstruction (iRP911). Detail on the reaction, given with identifiers of the type R-XXXX, can be found in the Additional file 2. [file 1752-0509-5-189-S6.PDF]

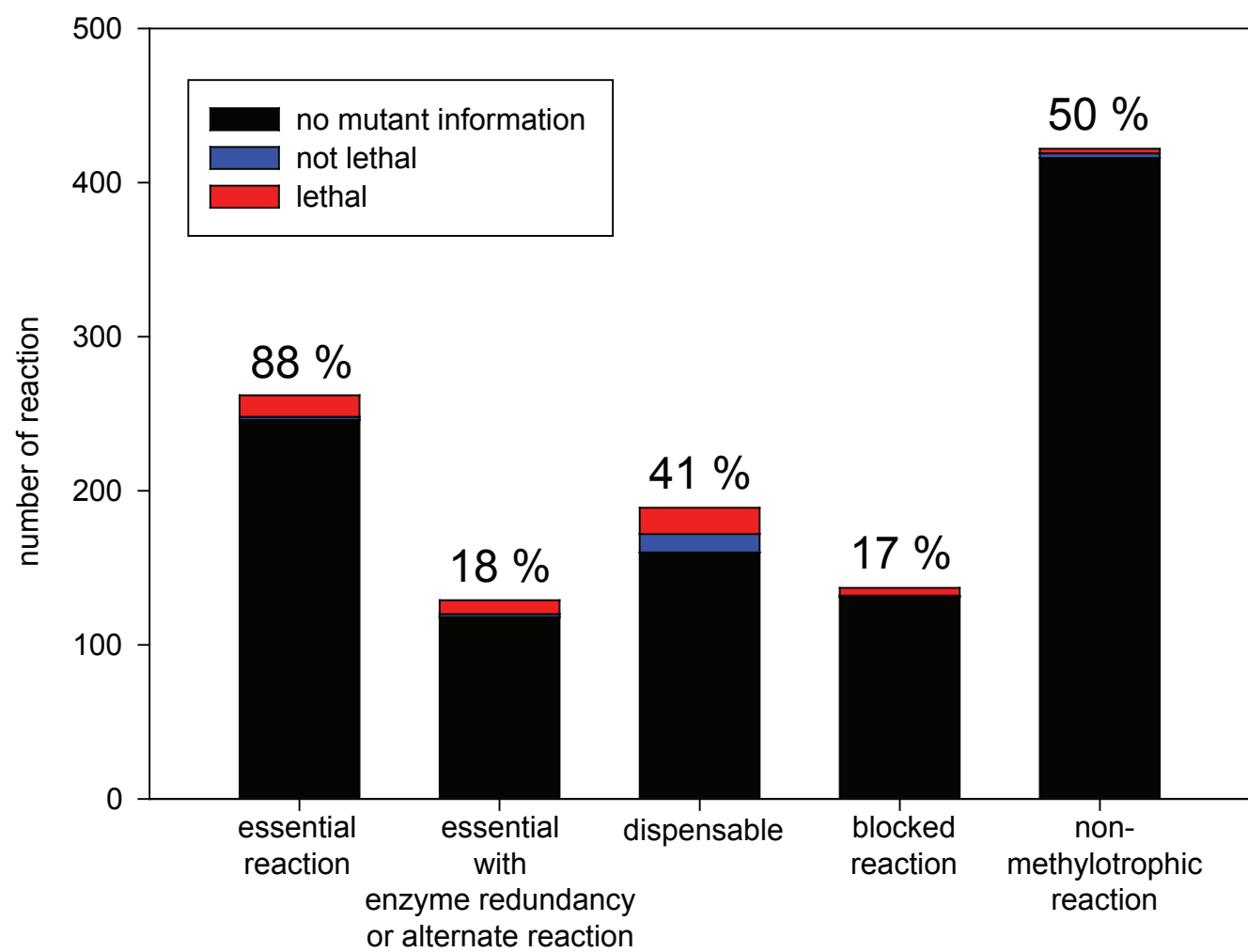

Supplement: Additional file 9 — Reaction essentiality in the methylotrophic network. The graph displays the essentiality or dispensability of reactions and showx the experimental evidence for gene essentiality. Reaction essentiality was analyzed using Minimal Cut Set calculation [42] applied to the set of EFMs [30] allowing biomass production from methanol. Fragility Coefficients (FCs) were calculated from the MCSs [42]. Reactions having a FC of 1 were identified as essential (red arrows), and reactions with a FC < 1 were considered as dispensable (blue arrows). The enzyme(s) catalyzing the network reactions are represented by boxes. The experimental phenotypes of mutants affected for these enzymes are displayed using a color code: red box: lethal phenotype, blue box: non-lethal phenotype, black box: no experimental data available. The accuracy of the model prediction is indicated upper the bar for each class of reaction. [file 1752-0509-5-189-S9.PDF]

Biological replicate 1

A1

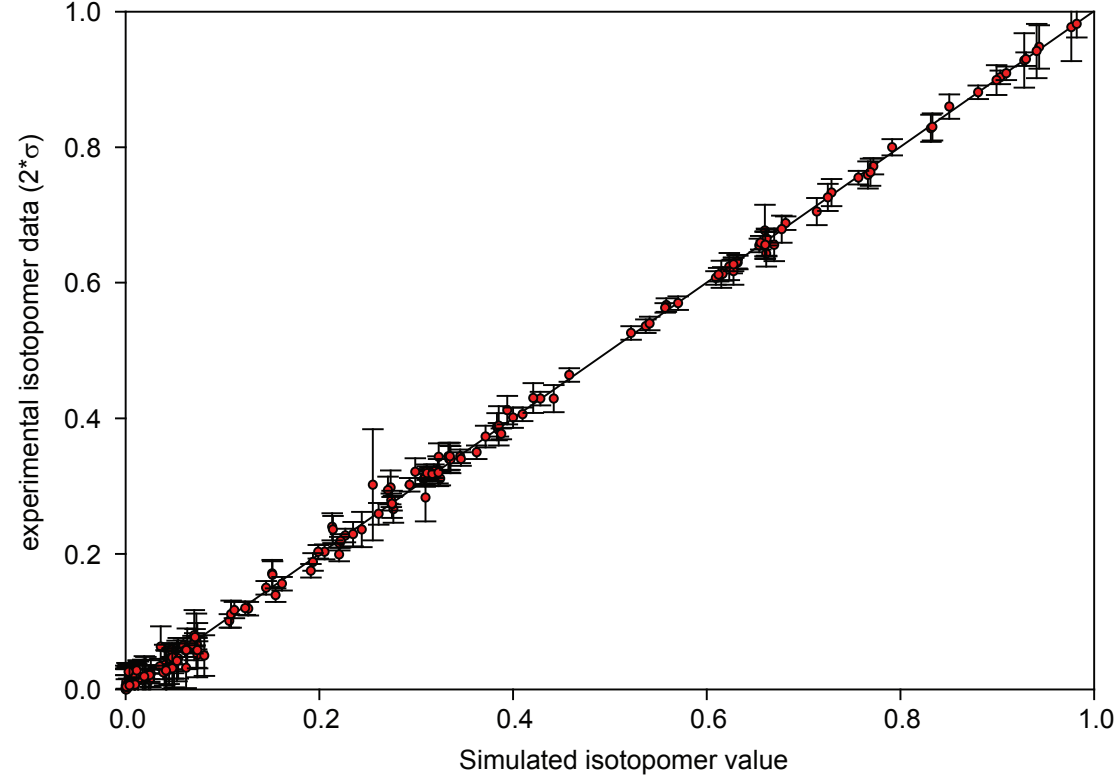

B1

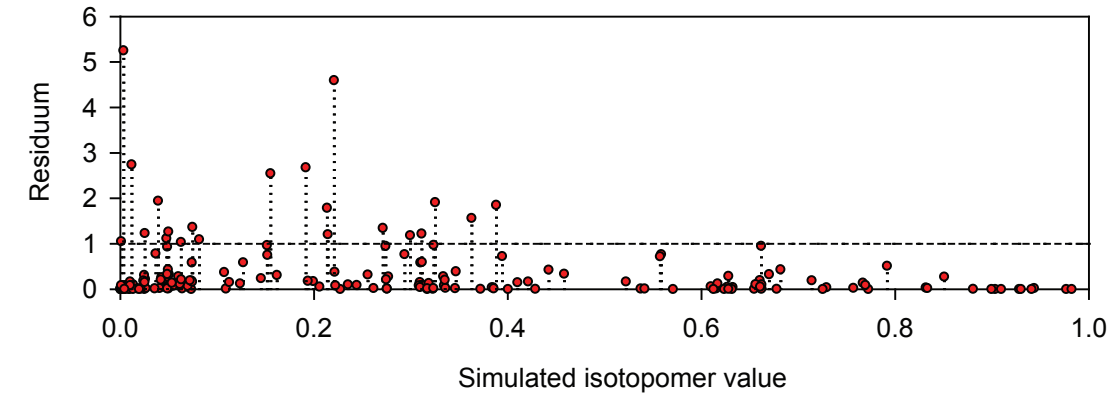

Biological replicate 2

A2

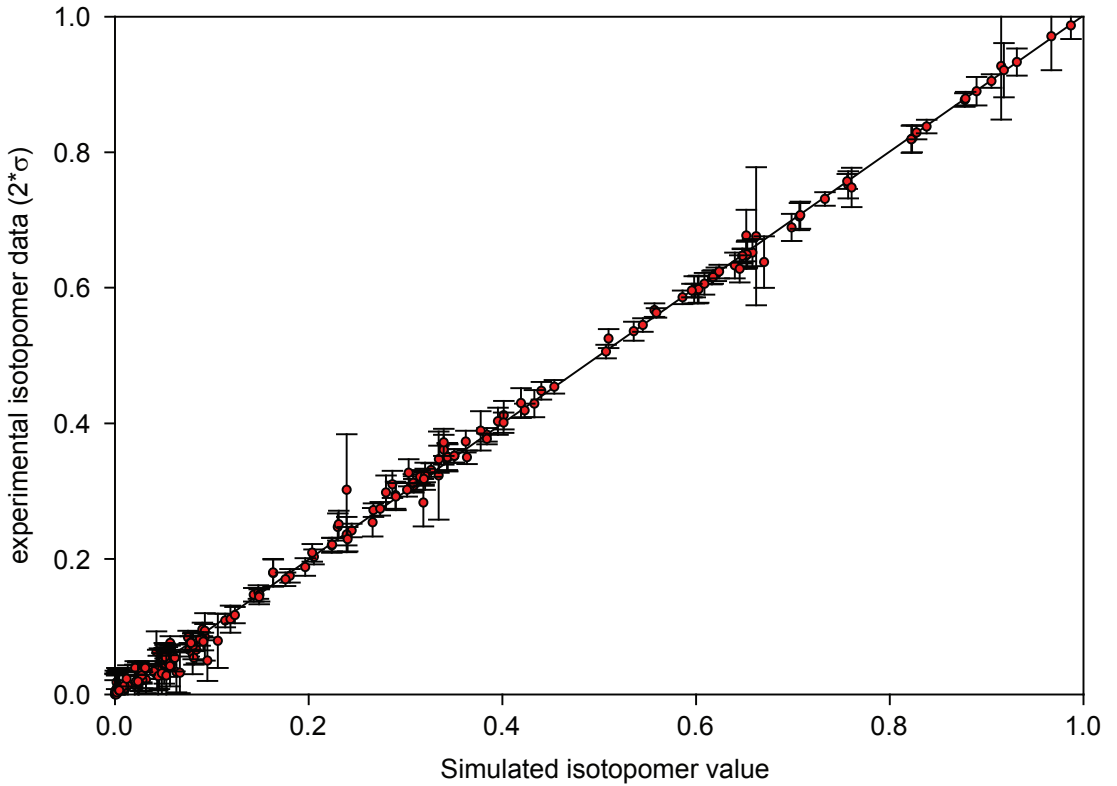

B2

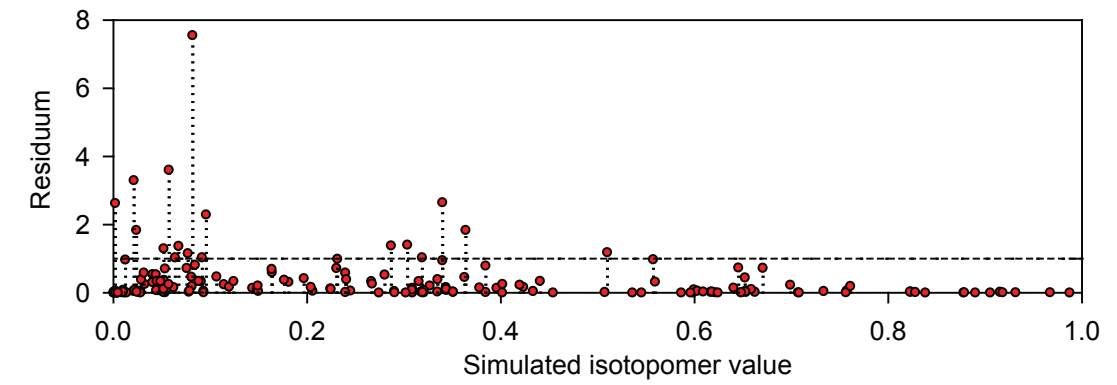

Biological replicate 3

A3

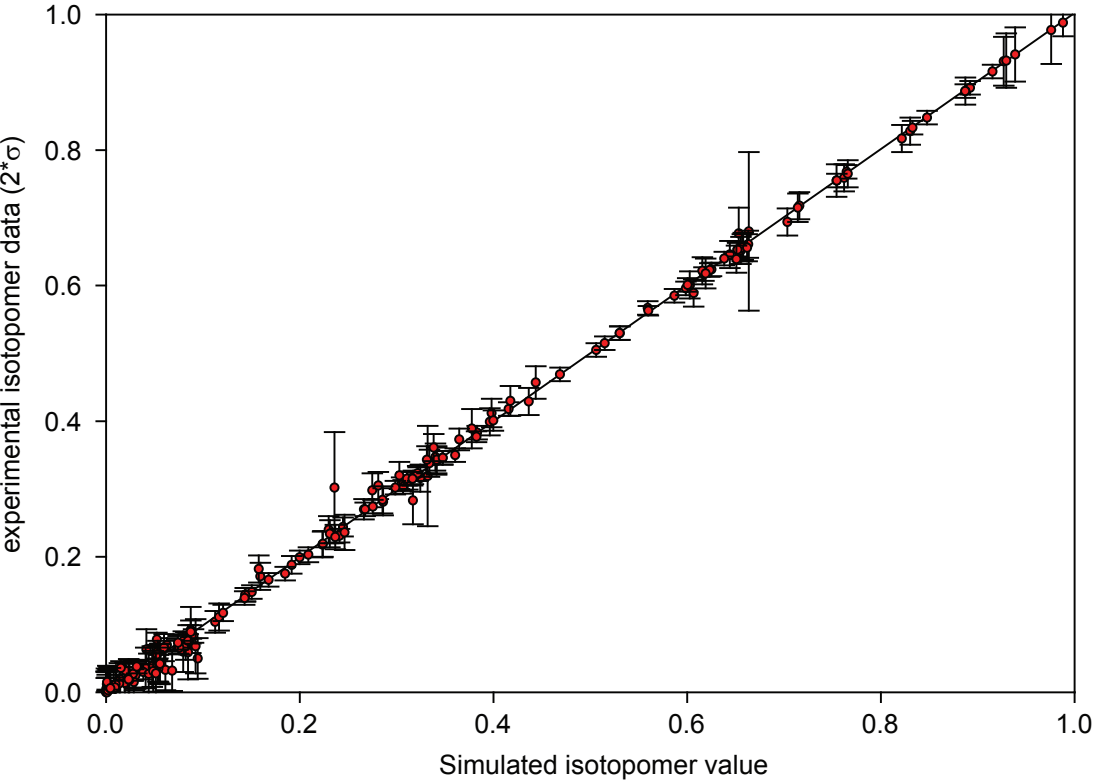

B3

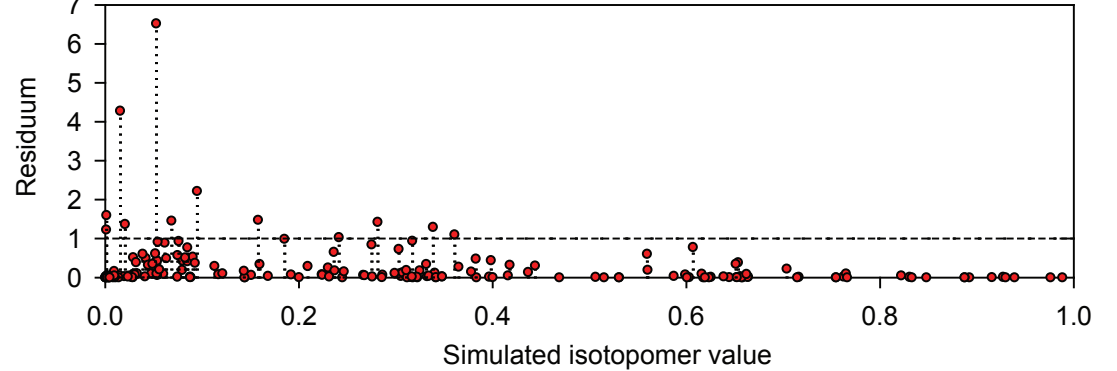

Supplement: Additional file 14 — Quality of isotopomer fitting. Comparison of experimental and collected isotopomer values for the three biological replicates. The isotopomer data include both LC-MS and 2D-NMR (HSQC and TOCSY) data. Flux calculation and fitting were performed using the software 13CFlux (Wiechert et al, 2001). A) Experimental values (+/- standard deviation) are plotted against theoretical values. B) Residuum of the calculated data. [file 1752-0509-5-189-S14.PDF]

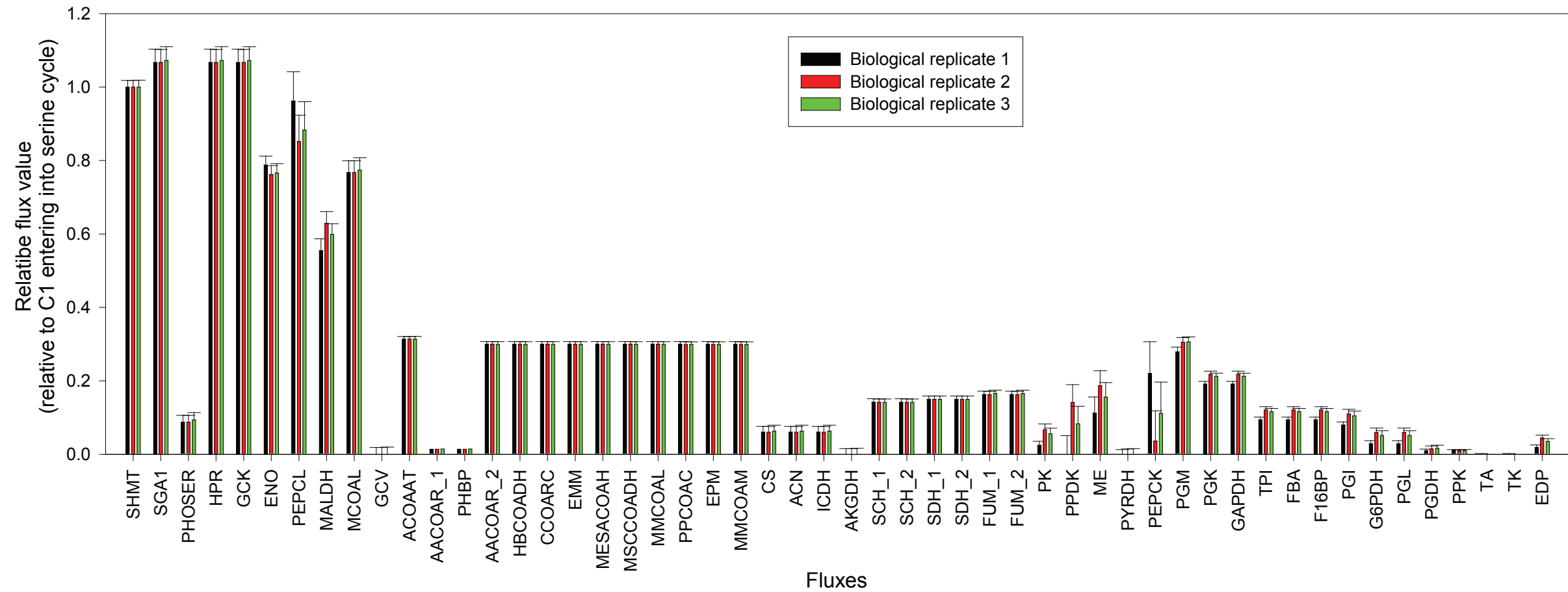

Supplement: Additional file 15 — Flux variability in the 3 biological replicates. Comparison of the flux distribution obtained for the three biological replicates. The flux calculation and the sensitivity analysis were performed using the software 13CFLUX (Wiechert et al, 2001). The fluxes were normalized by the flux of entry of the C1-units in central metabolism (SHMT: serine hydroxymethyltransferase). Flux distributions were found to be similar except slight changes through the C3/C4 interconversions (pyruvate kinase (PK), pyruvate dikynase (PPDK), malic enzyme (ME) and the phosphoenolpyruvate carboxykinase (PEPCK)), and through the Entner-Doudorof pathway. [file 1752-0509-5-189-S15.PDF]
